# Modeling Alkyl Aromatic Hydrocarbons with Dissipative Particle Dynamics

**DOI:** 10.1021/acs.jpcb.2c02048

**Published:** 2022-07-07

**Authors:** David J. Bray, Richard L. Anderson, Patrick B. Warren, Kenneth Lewtas

**Affiliations:** † The Hartree Centre, STFC Daresbury Laboratory, Warrington WA4 4AD, United Kingdom; ‡ Lewtas Science & Technologies Ltd., 246 Banbury Road, Oxford OX2 7DY, United Kingdom; ¶ School of Chemistry, The University of Edinburgh, Joseph Black Building, David Brewster Road, Edinburgh EH9 3FJ, United Kingdom

## Abstract

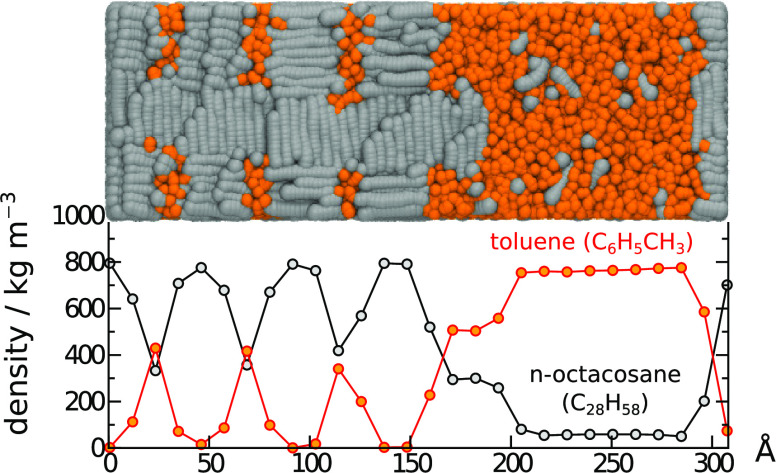

Building on previous work studying alkanes, we develop a dissipative particle dynamics (DPD) model to capture the behavior of the alkyl aromatic hydrocarbon family under ambient conditions of 298 K and 1 atmosphere. Such materials are of significant worldwide industrial importance in applications such as solvents, chemical intermediates, surfactants, lubricating oils, hydraulic fluids, and greases. We model both liquids and waxy solids for molecules up to 36 carbons in size and demonstrate that we can correctly capture both the freezing transition and liquid-phase densities in pure substances and mixtures. We also demonstrate the importance of including specialized bead types into the DPD model (rather than solely relying on generic bead types) to capture specific local geometrical constructs such as the benzene ring found in the benzyl chemical group; this can be thought of as representing subtle real-world many-body effects via customized pairwise non-bonded potentials.

## 1 Introduction

Alkyl aromatics are a series of very important chemicals in use worldwide. The shorter alkyl benzenes, such as toluene, xylene, ethyl benzene, etc., are found in solvents, fuel blending, adhesives, paints, and inks. Perhaps their most important uses are as chemical intermediates to make industrial important materials; for example, *p*-xylene is used as a precursor to make terephthalic acid, which then used to make polyethylene terephthalate (PET); *o*-xylene is used to make phthalic acid/anhydride, which can then be made into phthalate plasticizers; ethyl benzene can be converted into styrene, which can then be converted into polystyrene (PS); *m*-xylene can be converted into alkyd resins.^1^ The longer alkyl aromatics are equally important to today’s technology: they are precursors in the manufacture of surfactants that go into lubricating oils, cleaners, and detergents. For example, linear alkylbenzenes (LAB) are high-volume products and are used to make sulfonates (LABS) to be used in household detergents, dishwashing liquids, laundry liquids, laundry powders, and other household cleaners.^2,3^ It should be noted that linear alkyl groups are preferred because they biodegrade much more rapidly.^4^

There are several readily available atomistic molecular dynamics (MD) force fields with parameters for benzene and benzene-containing molecules; these include CHARMM27,^5,6^ AMBER ff19SB,^7^ etc., used for the study of proteins, lipids, DNA, and many small organic molecules and OPLS-AA, OPLS-CS,^8,9^ TraAPPE,^10,11^ etc., for hydrocarbons. To access longer time scales, coarse-grained (CG) molecular dynamics (CG-MD) models have been developed, which gain computational efficiency by reducing the level of molecular detail through combining atoms into CG beads. This approach has led to several CG parameterization efforts for benzene such as simple solvents^12,13^ and via SAFT approaches.^14^

Dissipative particle dynamics (DPD) provides an alternative coarse-graining approach, which has seen significant development in recent decades.^15^ In the DPD approach, CG beads can be used to represent fragments of a molecule, whole molecules, or collections of molecules.^16−18^ These DPD beads interact via soft potentials that incorporate chemical specificity. A pairwise, momentum-conserving thermostat is typically also included, which yields simulations in an NVT ensemble.^19^ The combination of these enables DPD to access much longer length and time scales than MD or CG-MD type approaches.

DPD models of small benzene-derived molecules (e.g., benzene, toluene, etc*.*) have been used to study properties such as interfacial tension,^20^ phase diagrams, and the octanol–water partition coefficient.^21^ However, in these models, the benzene-derived molecules are always a component (often dilute) of a more complex system, and the anhydrous neat properties of these molecules are not the focus of the study. Moreover to our knowledge, the behavior of the longer alkylbenzenes has not been considered by simulation, and as such, a systematic and transferable parameter set has not been developed.

In our previous work on alkanes,^22^ we demonstrated that a DPD model that incorporates the appropriate angular rigidity and bond stiffness can exhibit a freezing transition, and for example, we can observe the spontaneous formation of crystalline domains in melts and the precipitation of crystals out of solution. Using this approach, we were able to develop a set of DPD interaction parameters for alkanes at room temperature, which obtains the correct liquid or solid state across the wide range of carbon chain lengths from *n*-pentane (C_5_) to *n*-pentatricotane (C_35_). Furthermore, we were able to show that waxy alkanes (C_18_ onwards) precipitate out of solution (where *n*-heptane is the solvent) at concentrations that correspond to experimental observation.

In this work, by the careful determination of the bond and angle constraints of the benzene ring and in addition to the non-bonded interaction, we extend our DPD force field to capture the liquid- and solid-phase behavior of alkylbenzenes under ambient conditions corresponding to room temperature (298 K / 25 °C) and atmospheric pressure (1 atm). In the course of this parametrization, we show that to capture the chemistry accurately, we need to include “bespoke” arrangements of bead types to represent certain chemical structures in contradistinction to the transferable bead types commonly associated with DPD (Table 1). It appears that these bespoke arrangements are necessary to account for subtle real-world many-body effects and represent them in the pairwise interactions.

**Table 1:**
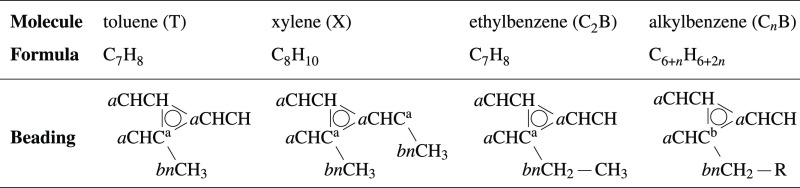
Examples of Chemical Structures Representable with the Current Model

In building the models, we consider the ambient conditions to be fixed, since to do otherwise would require extending the DPD methodology to incorporate the pressure and temperature dependence of these properties into the potentials as discussed previously in Bray et al.;^22^ accordingly, we leave this aspect to future work.

The remainder of the article is arranged as follows: In the Methods section, we outline the adopted CG model including details on the coarse-graining strategy, molecular architecture, and the crucial DPD interaction parameters used. We then give details of the simulation setup and summarize how solid waxes are identified from our simulations. The Results section presents the performance of the model in reproducing liquid densities and key behaviors associated with waxes (such as liquid–solid transitions and solubilities). Our conclusions are provided following the Results section.

## 2 Methods

### 2.1 Molecular Fragmentation Strategy

In our DPD model, each individual molecule is represented by a set of CG beads each of which represents some aspect of the local chemistry; these are “bonded” into a simplified structural representation of the original molecule. Each bead is defined to contain a complete set of bonded atoms (e.g., the chemical groups CH_2_CH_2_ in alkanes and CHCH in benzene provide bead types). These are designed to align with the standard chemical groups and enable (as far as possible) the set of standard beads to be transferable (with some exceptions as discussed later). Our beading strategy continues that begun by Bray et al.^22^ where *n*-alkanes were modeled by linear chains of DPD beads containing the groups CH_3_, CH_2_, and CH_2_CH_2_. As with our previous work, the CH_2_CH_2_ bead was used in preference to two CH_2_ beads when either choice was valid, meaning alkyl chains contain at most a single CH_2_ placed beside the terminating CH_3_ bead. We use the parameters from our previous model to describe the alkane chains present in the alkylbenzene molecules without modification, with the exception of the point of connection to the benzene ring. Here, we introduce a modified CH_2_ bead (denoted *bn*CH_2_; described in more detail below), which provides us an additional degree of freedom to develop the current model. This is chemically appropriate also as the first carbon is significantly influenced by the benzene ring.

Benzene is modeled by three identical beads, each comprising two carbons and connected into a bonded triangle, defined via the bead type *a*CHCH. Substituted ring arrangements consist of combinations of *a*CHCH and *a*CHC beads, where the latter allows for a side chain off the ring (note: *a*CC would also be valid but is not included in the study). For example, toluene contains two *a*CHCH beads and one *a*CHC bead. Additionally, as mentioned above, to allow it to deviate in behavior from the standard alkane group, the alkyl bead immediately adjacent to and attached to the benzene ring is given its own distinct bead type *bn*CH_3_ or *bn*CH_2_. This combination, comprising the benzene ring and first carbon off the ring, therefore represents the *benzyl* structural motif. We limit ourselves to these two bespoke bead types and did not consider a *bn*CH_2_CH_2_ being attached to the benzene ring as this could overly complicate the resultant angle distributions, making it difficult to adequately model using the simple DPD angle potentials. Note that we do not intend the *a*CHCH and *a*CHC beads to be used with chemistries that significantly perturb the benzene ring, such as the OH group in phenol, since we believe that this would likely require different bead types and further parametrization.

As there is already a huge diversity of molecular structures that comprise one or more alkane groups attached to a benzene ring, we do not consider structures with branched side chains such as isopropylbenzene, which would require a branched benzyl bead *bn*CH, structures with two side chains off one of the core benzene beads such as 1,2,3-trimethylbenzene, fused ring structures such as naphthalene, or structures with fused benzyl groups such as diphenylmethane in the current study. Nevertheless, bond lengths and bond angles described in the paper could be used to represent these molecules where indicated. Table 1 shows the representative schematics of the bead and bond configurations used for the various molecules in the present study.

### 2.2 Non-Bonded Interactions

The non-bonded interactions in the model are defined by the standard DPD short-range, soft, pairwise repulsions given by the pair potentials  (for *r_ij_* ≤ *R_ij_*), where β = 1/*k*_B_*T*, *r_ij_* = | r_*j*_ – r_*i*_ | is the separation between beads *i* and *j* located at r_*i*_ and r_*j*_, respectively, *A_ij_* is the repulsion amplitude, and *R_ij_* is the cut-off distance. To fix the cut-off distances, *R_ij_*, we note that different bead types contribute unequally to the molar volume of the molecules concerned; therefore, we follow the methodology previously successfully used for surfactants and the earlier DPD alkane model.^21−24^ We first assign *R*_*ii*_^3^ for different beads in proportion to the fragment (bead) molar volume using the Durchschlag and Zipper rules,^25^ taking the molar volume of a water bead (2 × H_2_O) as a reference. This fixes the cut-off distance *R_ii_* between DPD beads of the same type. Thereafter, we use a simple arithmetic mixing rule  to define the cut-off between dissimilar bead types.

The repulsion amplitudes, *A_ij_*, are fitted such that the experimental densities of pure alkylbenzenes at room temperature (298 K / 25 °C) and atmospheric pressure (1 atm) are reproduced by DPD simulations (as discussed in the Results section). For this current study, we adopt the interaction parameters for alkanes directly from Bray et al.^22^ and did not optimize these further. The remaining *A_ij_* values were fitted sequentially as described in the Results section. Tables S1 and S2 of the Supporting Information (SI) list the experimental densities and melting temperatures of molecules considered in this work. Table 2 presents the optimized set of *A_ij_* and *R_ij_* values for the model.

**Table 2:** Repulsion Amplitudes (*A_ij_*) and Cut-off Distances (*R_ij_*) between all Bead Pairs in the Model

bead *i*	bead *j*	*A_ij_*	*R_ij_*
*alkane*^a^			
CH_2_CH_2_	CH_2_CH_2_	19.5	1.0740
CH_2_CH_2_	(*bn*)CH_3_	25.9	1.0155^b^
CH_2_CH_2_	(*bn*)CH_2_	12.8	0.9995^b^
(*bn*)CH_3_	(*bn*)CH_3_	33.0	0.9570
(*bn*)CH_3_	(*bn*)CH_2_	19.2	0.9410^b^
(*bn*)CH_2_	(*bn*)CH_2_	5.0	0.9250
*benzyl*			
*a*CHCH	*a*CHCH	29.5	0.9700
*a*CHCH	*a*CHC	29.5	0.9540^b^
*a*CHC	*a*CHC	29.5	0.9380
*bn*CH_3_	*a*CHCH	24.5	0.9635^b^
*bn*CH_3_	*a*CHC	7.0	0.9475^b^
*bn*CH_2_	*a*CHCH	5.0	0.9475^b^
*bn*CH_2_	*a*CHC	9.5	0.9315^b^
*alkane–benzene*			
CH_2_CH_2_	*a*CHCH	25.9	1.0220^b^
CH_2_CH_2_	*a*CHC^a^	8.6^c^	1.0060^b^
CH_2_CH_2_	*a*CHC^b^	15.5^c^	1.0060^b^
CH_3_	*a*CHCH	33.0	0.9635^b^
CH_3_	*a*CHC^a^	11.0^c^	0.9475^b^
CH_3_	*a*CHC^b^	19.8^c^	0.9475^b^
CH_2_	*a*CHCH	19.2	0.9475^b^
CH_2_	*a*CHC^a^	6.4^c^	0.9315^b^
CH_2_	*a*CHC^b^	11.5^c^	0.9315^b^

a Same as Bray et al.^22^

b Cross term *R_ij_* obtained by combination rule.

c There are two bead types for the aCHC group: *a*CHC^a^ for methyl/ethyl side chain; *a*CHC^b^ for all other alkyl side chains.

### 2.3 Bonded Interactions

Molecules are assembled by connecting together the appropriate DPD beads using pairwise bonds, augmented by three-body angular potentials to confer additional rigidity to the structures. For the pairwise bonds we use a harmonic spring potential  where *K*_B_*^ij^* = 5000 *k*_B_*T* is the bond (spring) constant, *r_ij_* is the distance between bonded beads *i* and *j*, and *r*_0_^*ij*^ is the equilibrium bond length. We use this relatively large value for *K*_B_^*ij*^ since it prevents large bond length fluctuations from occurring compared to the more traditional lower values adopted in the DPD models. This results in better agreement in bond lengths between these models and atomistic simulations as discussed previously in Bray et al.^22^ Values for *r*_0_^*ij*^ are determined via molecular representations using the same method as described in Bray et al.^22^ (e.g., for the *a*CHCH–*a*CHCH bond using a model of benzene based on experimental bond lengths; see the SI). Table 3 lists the bonded interaction parameters used in this work.

**Table 3:** Rationalized Bonded Interaction Parameters in the Model

bead *i*		bead *j*	*K*_B_^*ij*^	*r*_0_^*ij*^
*alkane*^a^				
CH_2/3_	—	(*bn*)CH_2_	5000	0.30
CH_2_CH_2_	—	(*bn*)CH_2/3_	5000	0.35
CH_2_CH_2_	—	CH_2_CH_2_	5000	0.44
*benzyl*				
*a*CHC(H)	—	*a*CHC(H)	5000	0.39
*a*CHC	—	*bn*CH_2/3_	5000	0.35

a Same as Bray et al.^22^

Molecular rigidity is enhanced by the inclusion of an angular three-body potential between pairs of bonds. We adopt the same harmonic angular potential used by Smit and collaborators,^26,27^, where θ_*ijk*_ (in radians) is the angle between adjoining bonds and θ_0_^*ijk*^ is the equilibrium angle based on the chemical identities of *i*, *j*, and *k*. The θ_0_^*ijk*^ and *K*_A_*^ijk^* values for alkanes have been previously calibrated such that the appropriate length *n*-alkane freezes at room temperature (ee Bray et al.^22^). We set the remaining θ_0_^*ijk*^ based on molecular representations (SI). The angular spring constant, *K*_A_^*ijk*^ (in units rad^–2^), is optimized to capture the dominate peak in the angle distribution as calculated from atomistic simulations (as described in the SI).

**Figure 1:**
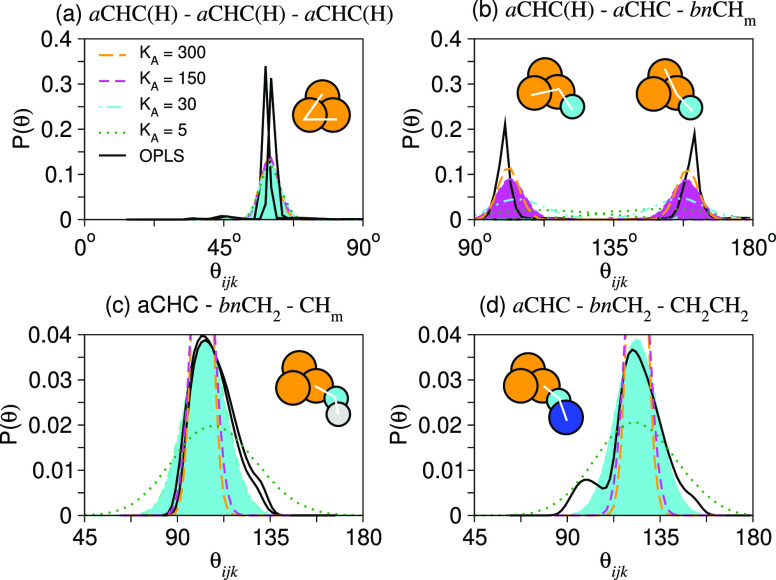
Comparison of the angle distribution function *P*(θ) obtained from DPD (dashed/dotted) and MD (solid). The shaded curve gives the accepted distribution used in this work.

Figure 1 shows the effect of varying *K*_A_^*ijk*^ on the angle distributions compared to those calculated from atomistic simulations and highlights the chosen value for each case. Table 4 lists the angle potential parameters used in this work. Pragmatic choices were made aromatic bond angles, such as *a*CHC(H)-*a*CHC(H)-*a*CHC(H) or *a*CHC(H)-*a*CHC(H)-*bn*CH_m_, to keep the vibrational frequency small compared to the DPD time step. Nevertheless, the resultant angle distribution is tight enough for steric differences to come through, for example, the model displays differences between the isomers of xylene, which would be hidden by use of a lower angular stiffness such as *K*_A_^*ijk*^ = 5.

**Table 4:** Rationalized Angular Potential Parameters in the Model

bead *i*		bead *j*		bead *k*	*K*_A_^*ijk*^	*θ*_0_^*ijk*^
*alkane*^a^						
(*bn*)CH_2/3_	—	CH_2_CH_2_	—	CH_2/3_	150	180°
(*bn*)CH_2/3_	—	CH_2_CH_2_	—	CH_2_CH_2_	150	166°
(*bn*)CH_2/3_	—	CH_2_	—	CH_2/3_	150	100°
(*bn*)CH_2/3_	—	CH_2_	—	CH_2_CH_2_	150	125°
CH_2_CH_2_	—	CH_2_CH_2_	—	CH_2_CH_2_	70	180°
CH_2_CH_2_	—	CH_2_	—	CH_2_CH_2_	150	146°
*benzyl*						
*a*CHC(H)	—	*a*CHC(H)	—	*a*CHC(H)	30	60°
*a*CHC(H)	—	*a*CHC	—	*bn*CH_2/3_	150	160°
*bn*CH_2/3_	—	*a*CHC	—	*a*CHC(H)	150	100°
*a*CHC	—	*bn*CH_2_	—	CH_2/3_	30	102°
*a*CHC	—	*bn*CH_2_	—	CH_2_CH_2_	30	124°

a Same as Bray et al.^22^

### 2.4 General Conditions of the Simulations

DPD simulations were performed using the dl_meso 2.7 simulation package.^28^ Periodic boundary conditions were assumed in all three spatial dimensions. All beads have a reduced mass of 1 and are charge neutral. It is convenient to set the DPD unit of length *r*_c_ = 5.65 Å as in our previous works.^22,23^ Although water does not feature in the present study, this corresponds to treating water (H_2_O) supramolecularly with a mapping number *N*_m_ = 2, assigning the water bead repulsion range *R_ii_* = *r*_c_ , and assuming the reduced water bead density under ambient conditions (see below) is ρ*r*_c_^3^ = 3. We thermostat the model with the standard DPD pairwise random and dissipative forces^15,29^ with damping coefficient *γ* = 4.5, and range 1.1 *r*_c_ just above the maximum *R_ij_* used for the conservative forces. The thermal scale in DPD units is by convention set at *k*_B_*T* = 1. We note that by making the *A_ij_* and *R_ij_* parameters temperature-dependent (i.e., on the real-world temperature), in principle this convention can always be retained. Constant pressure simulations were performed using the Langevin piston implementation of Jakobsen using barostat parameters τ_p_ = 2.0 and γ_p_ = 5.0.^30^ The combination of *k*_B_*T* = 1 and barostat target pressure *P* = 23.7 (in DPD units) furnishes our definition of “ambient conditions” and corresponds to water beads with repulsion amplitude *A_ii_* = 25 at the above reduced water bead density. A time step of Δ*t* = 0.01 was adopted for all simulations, and data was collected every 10 DPD time units (10^3^ time steps) following equilibration. The analysis code ummap was used to analyze simulation trajectory files.^31^

### 2.5 Pure Substances and Liquid–Liquid Mixtures

To simulate pure substances and liquid–liquid systems, molecules were initially configured in random orientations on a cubic lattice that spans the box dimensions. For the optimization of *A_ij_* and to study liquid–liquid mixtures, simulations were run for a total of 2 × 10^3^ DPD time units, where data was collected after the initial 10^3^ DPD time units. This timescale was deemed sufficiently long for final equilibrium states to emerge. For a representative subset of molecules, simulations were also undertaken in larger box sizes to test for finite-size effects (none were found). The SI provides details on system sizes and sampling times used.

### 2.6 Solubility Studies of Binary Mixtures Containing Long Alkanes

Following the setup procedure outlined in Bray et al.^22^ mixtures of solvents (i.e., T, B, C_2_B, *n*-C_7_, *n*-C_15_) and a long alkane (i.e., *n*-C_28_, *n*-C_32_) were started in a fully segregated arrangement. Here, a box of size 60 × 20 × 20 DPD units was divided into two subvolumes (along the long axis) with the relative volumes fixed by the bead fraction, and each of these subvolumes was filled by randomly placed molecules of the desired type. Each box contained a total of ∼72,000 beads, initially leading to a reduce density of 3, which adjusted during NPT simulation. We have found that this setup allows solid precipitation (of the long alkane) to occur even at low mole fractions just above the solubility limit. Half of the beads were assigned to the solvent, and the other half was assigned to the long alkane. This ensured that for each system studied, the long alkane was at concentrations well above the solubility limit.

### 2.7 Identifying Waxes

The system is said to have solidified into a wax if it has high structural order and low mobility. To measure the degree of structural order, a unit vector **n**_*i*_ is defined for each molecule as the normalized vector separation between the beads at the ends of the alkyl side chain of the molecule (e.g., the first non-benzyl bead and final “tail” bead of the alkyl chain); this unit vector is oriented for definiteness and without loss of generality such that **n**_*i*_ · **e**_*z*_ ≥ 0, where **e**_*z*_ is the unit vector directed along the *z* axis. A local director (vector) **D**_*i*_ is then defined for each molecule by averaging the orientation vectors **n**_*i*_ for molecules with midpoints lying within a sphere of radius 5 *r_c_* (∼28 Å) of the midpoint of the target molecule. Finally, for each molecule, cos ϕ_*i*_ = **n**_*i*_ · **D**_*i*_/ | **D**_*i*_| is computed, and in terms of this, an orientational order parameter based on the usual Legendre polynomial is extracted: *S* = ⟨*P*_2_( cos ϕ)⟩ = ⟨(3cos^2^ϕ – 1)/2⟩. The order parameter obeys 0 ≤ *S* ≤ 1, with *S* ≈ 0 in an isotropic liquid and *S* → 1 as the molecules become perfectly aligned; the material is said to exhibit orientational order when *S* ≳ 0.5.

We use the mean square displacement (MSD) to assess molecular mobility. This is calculated from the bead coordinates as ⟨|**r**_*i*_(*t* + Δ*t*) – **r**_*i*_(*t*)|^2^⟩, where **r**_*i*_(*t*) is the position of the *i*th bead at time *t* after “unwrapping” the periodic boundary conditions. For concreteness, we report the MSD at Δ*t* = 500 DPD time units (hereafter MSD@500).

### 2.8 Estimating Solubility

For binary solvent + solute mixtures, the solute should precipitate out of the solution when the total added amount goes above the solubility limit for that solvent. This corresponds to the presence in the equilibrium phase diagram of a two-phase region in which a saturated solution at the solubility limit coexists with the precipitated material. Thus, to obtain an estimate for the solubility limit, we simply need to find the solvent-rich phase in a simulated phase coexistence and measure its composition. To this end, we adopt a method developed by Anderson et al.^21^ to calculate the octanol–water partition coefficients.

To do this, we measure from the simulation the time average local concentration of each molecule by dividing the simulation box evenly along the long axis into slabs of thickness ≈ 2 *r*_c_ and computing the concentration for all components in each slab. Next, the gradient profile is calculated, and the bulk phase domains are identified as the contiguous regions where the absolute gradient is less than a cut-off that we set equal to the standard deviation of the set of calculated gradient values. Note that this may result in more than two “bulk” phases being identified due to grain boundaries in the solid phase caused by changes in molecule orientation and packing of the long alkane. Nevertheless, the solvent-rich phase is always the one containing the highest concentration of solvent. Having identified the position of this phase, the average concentrations in this phase domain are then extracted and converted to weight fractions, *wf_i_*, and then volume fractions, ϕ_*i*_, using
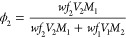
1

Here, *V_i_* is the molar volume, and *M_i_* is the molar mass of each molecular component *i*, where solvent is *i* = 1 and solute *i* = 2.^32^

## 3 Results

### 3.1 Liquid Densities for Benzene and Benzene + Alkane Mixtures

**Figure 2:**
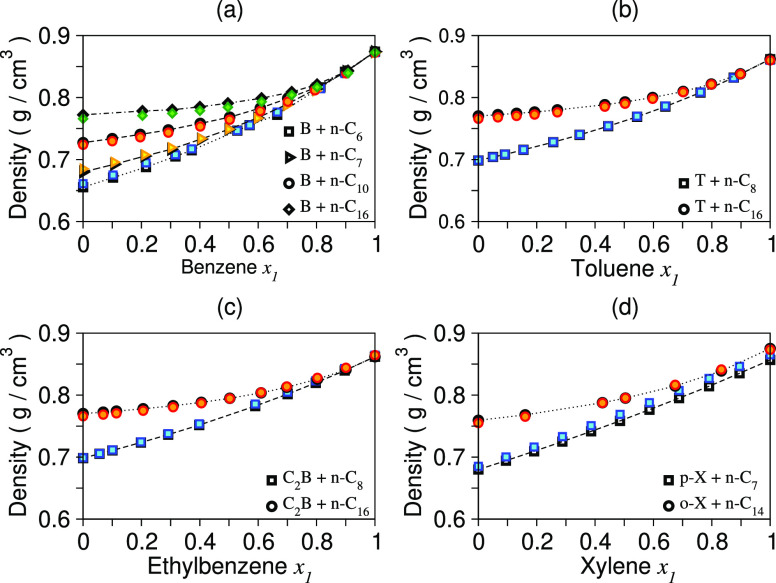
Density as a function of mole fraction for binary liquid mixtures. Dashed lines with black points are for experimental values, and colored points are for DPD model mixtures.

Repulsion amplitudes, *A_ij_*, for benzene (*a*CHCH beads self-interaction) and between benzene and alkanes (*a*CHCH interaction with CH_3_,CH_2_, and CH_2_CH_2_ beads) were obtained by matching the density for benzene, and four mixtures of benzene (B) + alkane (*n*-C_n_) using the experimental data in Teja and Rice.^33^ We achieved good agreement between the model and experimental densities, obtaining a difference of only 0.15% for benzene. While across the range of concentrations of benzene + alkane mixtures, we obtained root mean square deviation (RMSD) of 0.0037 (*R*^2^ = 0.997) for benzene + hexane (B + *n*-C_6_), 0.0026 (*R*^2^ = 0.998) benzene + heptane (B + *n*-C_7_), 0.0042 (*R*^2^ = 0.992) benzene + decane (B + *n*-C_10_) and 0.0055 (*R*^2^ = 0.968) benzene + hexadecane (B + *n*-C_16_), respectively. Figure 2a shows the comparison between the model prediction and experiment for different mole fractions of benzene for the benzene-alkane mixtures studied.

### 3.2 Evidence for the Need to Capture Differences in Ring Behavior

It may seem reasonable to assume that with the above parameters specified, it is then possible to build the other members of the alkylbenzene family, e.g., toluene, ethylbenzene, hexylbenzene, etc., and get the liquid density correct. These molecules can be built by attaching alkane side chains off the benzene ring via first swapping the relevant *a*CHCH bead to *a*CHC, then attaching the alkane chain, starting with a CH_2_ (or CH_3_ for a single methyl group such as toluene) followed by intermediate CH_2_CH_2_ beads and terminated by either by a CH_3_ bead for an even-length chain or by a CH_2_-CH_3_ bead for an odd-length chain. Some schematics of such molecules are shown in Table 1. In the above cases, the *a*CHC bead has the same interaction parameters as benzene.

**Figure 3:**
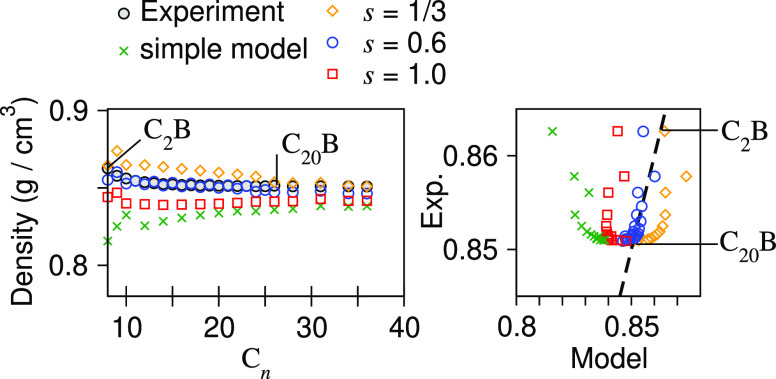
Liquid-phase densities of alkylbenzenes (C_*n*_B) for various model versions including the final choice *s* = 0.6. The dashed line in the right-hand plot signifies perfect agreement with experiment.

To test this approach, we built models of toluene (T), the symmetrically substituted 1,3,5-trimethylbenzene known as mesitylene (M), and the alkylbenzenes (C_*n*_B): ethylbenzene (C_2_B) through to triacontylbenzene (C_36_B) (SI). We calculated the model predictions for the densities for these molecules as described in the previous paragraph and found poor agreement with the experiment, producing a RMSD of 0.03704 but with *R*^2^ = – 32.4. The crosses (green) of Figure 3 show how the densities predicted using this “simple” initial model deviate from the experimental values shown as solid circles and squares. As it is set up, there is excellent agreement for benzene, but it is not possible to fit with similar accuracy toluene^34^ and mesitylene,^35^ where the disagreement between the model and the experiment is 6.27 and 14%, respectively (SI). This agreement improves for alkylbenzenes but still remains around the 2% mark at longer alkyl lengths (even at side-chain lengths of C_18_). Furthermore, the alkylbenzene model densities increase monotonically with increasing carbon side-chain length (more like a modified alkane family) rather than decreasing as actually seen in the experiment; this is the cause of the negative *R*^2^ value. Hence, we conclude that some of the parameters must be different from those needed for benzene or alkanes.

An obvious starting place would be to assume that the interactions of an *a*CHC bead are simply different to those of an *a*CHCH bead. However, this turns out to be insufficient as when we attempted to change only the *a*CHC···*a*CHC bead pair interaction (dropping its value down to as low as *A_ij_* = 1 ), we found only a minor improvement on the density for mesitylene and the error remained above 11%. Hence, we found that changing the ring parameters simply shifts the density and does not address the trend behavior with the side-chain length of the alkybenzenes. Instead, the bonded CH_2_ or CH_3_ bead adjacent to the *a*CHC needs to treated differently to standard alkanes, i.e., it should be a “specialized” bead type that captures the unique geometry and reflects the benzyl nature of the chemistry, which we will denote using the *bn* prefix.

### 3.3 Liquid-Phase Densities of Methylbenzene Derivatives

Parameters for methylbenzene were determined by matching the density of mesitylene, *o*-xylene, *p*-xylene, *m*-xylene, and toluene to set the interactions between *bn*CH_3_, *a*CHC, and *a*CHCH. Experimental data was obtained from Asfour et al.,^34^ Chevalier et al.,^36^ and Al-Kandary et al.^35^ The use of an *a*CHC bead to represent a benzene bead with an attached side chain allows the interaction parameters to be different for *bn*CH_3_···*a*CHC and *bn*CH_3_···*a*CHCH. For simplicity, we kept the repulsion amplitudes *A_ij_* for *a*CHC and *a*CHCH the same as benzene. However, tests indicate that deviating from this assumption has only minor affects, as discussed in the preceding section. Similarly, benzyl CH_3_ behaves as alkyl CH_3_ with respect to alkanes. Despite the difference in *R_ij_* values for *a*CHCH and *a*CHC, we kept the geometry of the benzene ring unchanged and independent of its constituent beads. In the real molecular representation (e.g., the CG representation of the aromatic ring of benzene, toluene, xylene, etc.), the actual variation in bond length and angle is small (within 0.11 Å and 5°, respectively). The benzyl *bn*CH_3_ interactions with the benzene ring are fitted by first obtaining *bn*CH_3_···*a*CHC repulsion amplitude using density data from mesitylene and then fitting *bn*CH_3_···*a*CHCH interaction using the data of toluene to obtain an agreement in densities between model and experiment of 0.01 and 0.22%, respectively. The performance of these parameters were confirmed against the variants of xylene, obtaining −0.98, −0.01, and 0.22% for *p*-xylene, *m*-xylene, and *o*-xylene, respectively, so that *m*-xylene gives the best agreement while *p*-xylene gives the worst (yet still being close). This is possibly due to similarity in methyl placement between mesitylene and *m*-xylene, which *p*-xylene does not share, and better agreement could be obtained by fitting it against structural isomers of mesitylene such as 1,2,4-trimethylbenzene and 1,2,5-trimethylbenzene. Note there are two ways to assign beads to the structure of *o*-xylene since the methyl groups are attached to adjacent carbons in the ring: the first is to have each methyl attached to a separate *a*CHC bead as discussed here; the second is to attach both methyl groups to the same *a*CC bead (not discussed).

**Figure 4:**
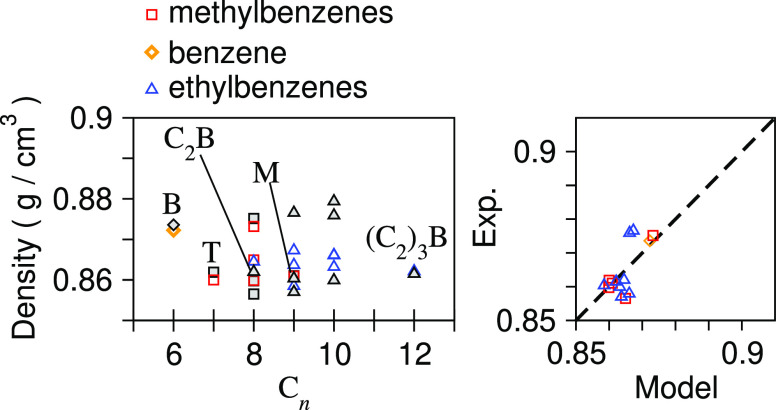
Liquid-phase densities for benzene, methylbenzene, and ethylbenzene derivatives. Black points are for experimental values, and colored points are for DPD models.

Figure 4 shows a comparison between the model densities and experimental values for these small benzyl molecules. These models are able to capture differences between the different molecules and isomers, which would not be possible if a simpler model was used.

### 3.4 Liquid-Phase Densities of Binary Mixtures of Methylbenzene and Alkanes

To obtain the repulsion amplitudes between the *a*CHC and alkane beads, we modeled four binary mixtures: toluene + *n*-octane (T + *n*-C_8_); toluene + *n*-hexadecane (T + *n*-C_16_); *p*-xylene + *n*-heptane (*p*-X + *n*-C_7_), and *o*-xylene + *n*-tetradecane (*o*-X + *n*-C_14_) using the experimental data from Asfour et al.,^34^ Yang et al.,^37^ and Chevalier et al.^36^ Here, we assumed that the *bn*CH_3_ bead present in the aromatics interacts with the alkanes (e.g., CH_2_CH_2_, CH_2_, and CH_3_) in the same way as the alkyl CH_3_.

We found that the remaining interactions of the *a*CHC bead with the alkane beads should not share the same values as the *a*CHCH bead, for by doing so would underpredict the density by up to 1.39% (T + *n*-C_16_) and 1.56% (T + *n*-C_8_). Instead, we found that a good fit can be obtained by scaling the relevant repulsion amplitudes by a factor *s* = 1/3 (i.e., *A_ij_* → *s A_ij_* for the relevant bead pairs). These parameters are marked aCHC^*a*^ in Table 2. Across the range of concentrations, we obtained RMSD values of 0.0031 (*R*^2^ = 0.988) for T + *n*-C_16_, 0.0005 (*R*^2^ = 0.999) for T + *n*-C_8_, 0.0095 (*R*^2^ = 0.971) for *p*-X + *n*-C_7_, and 0.0019 (*R*^2^ = 0.993) *o*-X + *n*-C_14_, respectively. Figure 2b,d compares the model density predictions with experiment for these mixtures.

### 3.5 Liquid-Phase Densities of Ethylbenzene Derivatives and Mixtures with Alkanes

A similar procedure was used to obtain the repulsion amplitudes between the benzyl bead *bn*CH_2_ and the *a*CHC(H) bead for side chains of a carbon length of 2 (i.e., −*bn*CH_2_-CH_3_). Experimental data was obtained from Asfour et al.^34^ and Dreisbach.^38^ The *bn*CH_2_···*a*CHC repulsion amplitude was obtained using density data from 1,3,5-triethylbenzene, and then the *bn*CH_2_···*a*CHCH repulsion amplitude was fitted using the data of ethylbenzene to obtain an agreement in densities between model and experiment of −0.07 and −0.22%, respectively. The *a*CHC···CH_3_ interaction is the same as used above. We validated these parameters by calculating the pure densities for the structural isomers of diethylbenzene, obtaining −0.95, −0.38, and 1.11% for *p*-diethylbenzene, *m*-diethylbenzene, and *o*-diethylbenzene, respectively. We also tested against structural isomers of ethyltoluene (a molecule containing both the *bn*CH_2_ and *bn*CH_3_ bead) and obtained −0.78, 0.22, and 1.06% for *p*-ethyltoluene, *m*-ethyltoluene, and *o*-ethyltoluene, respectively.

From this parameterization, we have sufficient information to explore liquid mixtures of ethylbenzene and alkanes. Specifically, we have explored binary mixtures of (i) ethylbenzene + *n*-octane (C_2_B + *n*-C_8_) and (ii) ethylbenzene + *n*-hexadecane (C_2_B + *n*-C_16_), and compared them to experimental data taken from Asfour et al.^34^ Again, our simulated results perform well when compared to the experimental data: we achieve RMSD values of 0.00223 (*R*^2^ = 0.998) for C_2_B + *n*-C_8_ and 0.00272 (*R*^2^ = 0.991) for C_2_B + *n*-C_16_, respectively. Figure 2c shows the comparison between the model density predictions and the experiment for these mixtures.

### 3.6 Evidence for the Effect of the Alkyl Side Chain on the Benzene Ring

At this point, we now appear to have the parameters for all of the DPD bead types required to calculate the densities of the alkylbenzene family, from propylbenzene (C_3_B) up to triacontylbenzene (C_30_B). When comparing the calculated densities against the experimental data from Dreisbach,^38^ these results fell within 2% of the experimental values (see the SI for individual data). However, the overall fit of the date gives an RMSD value of 0.00802 and *R*^2^ = – 5.14, which indicates that the trend was poor (see points of *s* = 1/3 in Figure 3 (right), where there is a clear bend away from the dashed line that indicates perfect agreement). Note that while the agreement is poor, this is still better than the simple model described earlier but worse than assuming all densities are take the mean values (see points of the simple model in Figure 3).

### 3.7 Liquid Densities of Longer-Chain Alkylbenzenes

As a consequence, we chose to re-parameterize the *a*CHC···alkane interactions, making them more repulsive, and instead use a scale factor of *s* = 0.6 compared to the *a*CHCH··· alkane interactions. This resulted in the parameters marked aCHC^*b*^ in Table 2. These changes result in a significantly improved fit with a maximum error of less than 0.5% per C_*n*_B (*n* > 2), an overall RMSD of 0.00233, and *R*^2^ = 0.999. However, when we try the new parameters with ethylbenzene (C_2_B), we underestimate the density by −0.86% compared to the experiment, which is far worse than the fit obtained using the *s* = 1/3 parameters. This suggests that the *a*CHC bead type from ethylbenzene should be treated differently from that needed for C_*n*_B (*n* > 2).

Figure 3 shows how well the alkylbenzene family (*n* > 2) fits the experimental data when using scale factor *s* = 1.0 (as used for benzene), 0.6 (as refined for alkylbenzene), and 1/3 (as used for methyl/ethylbenzene) and in the simple model. Here, we can see that the models with *s* = 0.6 fit much better than the others (raw data given in the SI).

We believe the requirement for the two variations of the *a*CHC beads (i.e., *a*CHC^a^ and *a*CHC^b^) is due to the severe overlap that occurs between the bonded beads in the DPD coarse-grained model. Here, the effective distance of non-bonded interaction is of the order *r_c_* = 5.65 Å (as suggested by *R_ij_* values), but the equilibrium bond lengths are typically ≲ 0.5 *r_c_*, which means that when another non-bonded bead moves close to one of these beads, it is subjected to the combined repulsion from the bead in question and its nearest bonded neighbors (in atomistic models, this effect is weaker or avoided since the bond lengths are typically much bigger than the non-bonded interaction cut-off distance). When the repulsion amplitudes for the first/second neighbor beads are large (such as for the methyl group), the contribution to the overall repulsion is stronger and the combined effect is more pronounced. Thus if the net repulsion should be similar between common groups (e.g., *a*CHC···CH_3_) the repulsion amplitudes for these groups must compensate those from the surrounding neighbors. This can be thought of as effectively capturing the many-body contributions of the surrounding bonded particles within a pairwise potential model.

### 3.8 Freezing Transition of Pure Alkylbenzenes

**Figure 5:**
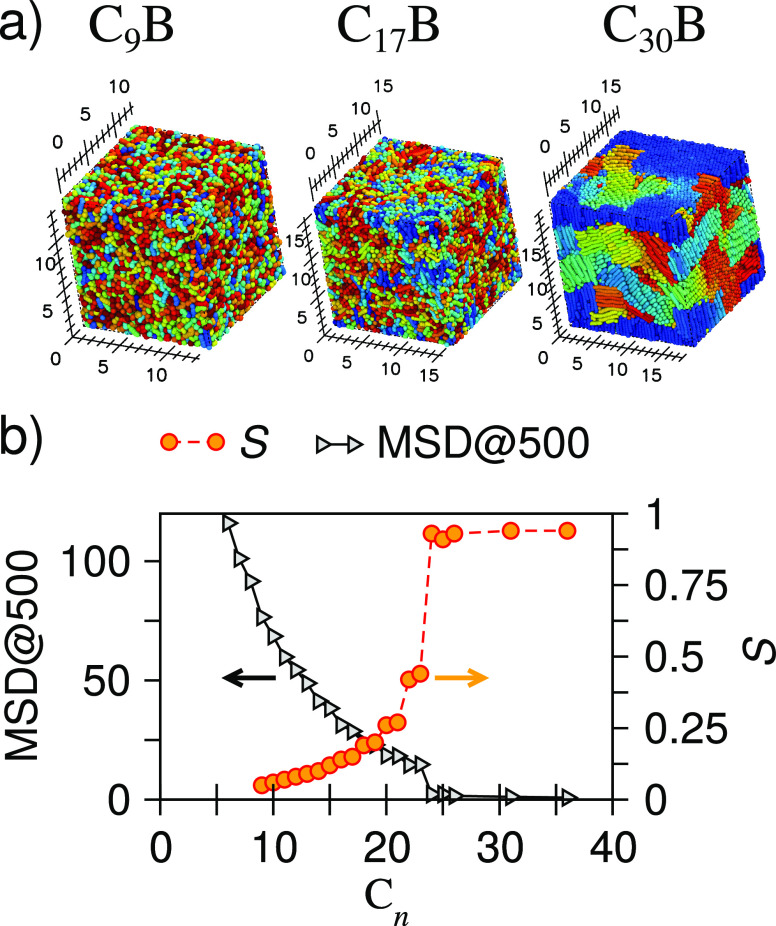
Physical state of alkybenzene (C_*n*_B) family: (a) snapshots of the system, (b) mean square displacement (MSD@500), and orientational order parameter *S*.

We next turn our attention to the freezing transition of pure alkylbenzenes. Experimentally, it is found that at room temperature freezing occurs between C_15_B to C_16_B (i.e., the former is liquid at room temperature, whereas the latter is a waxy solid). Adopting the parameterization we have developed in the present study thus far, we test the capability of the models to reproduce this. The final physical state of the simulated system is assessed by measuring the MSD at 500 time units and orientational order parameter, *S*. Results are shown in Figure 5. When the MSD sharply drops toward zero, the system is deemed to have solidified. Figure 5 shows that this takes place between C_17_B and C_18_B, two units longer in carbon chain length than expected experimentally. The high value of *S* indicates that crystalline order also prevails, and this is verified by visual inspection (shown for C_30_B in Figure 5a).

To test for finite-size effects, we examined three different simulation box sizes for the systems C_15_B to C_18_B consisting of 600, 1200, and 2400 molecules, respectively. We found no significant differences in the results (SI). We additionally determined that the liquid nature of C_17_B remains when simulations are significantly extended (up to 10^5^ DPD time units) beyond our standard protocol.

### 3.9 Differences in the Solubility of Waxy Alkanes in a Range of Solvents

Starting from an initially segregated random state (i.e., each molecule type placed randomly in each half of the box), we were able to estimate the solubility of *n*-C_28_ and *n*-C_32_ at room temperature in the solvents benzene, toluene, mesitylene, and ethylbenzene. Reference experimental data was taken from Haulait-Pirson et al.^32^ For comparison, we also measured the solubility of alkane mixtures *n*-C_15_ + *n*-C_20_, *n*-C_7_ + *n*-C_28_, and *n*-C_7_ + *n*-C_32_.

**Figure 6:**
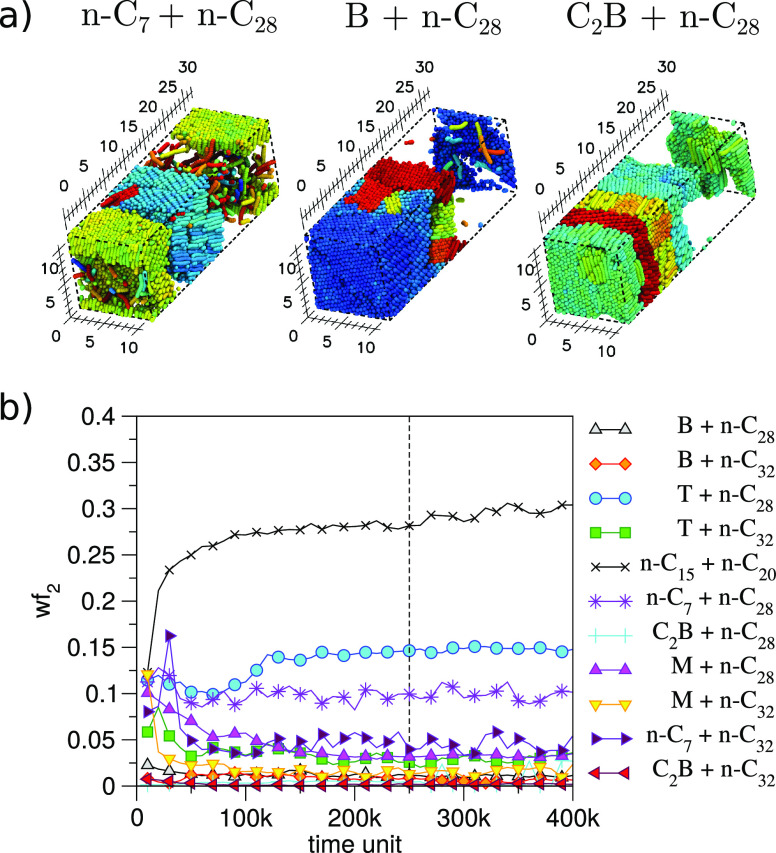
Solubility of waxy alkanes in aromatic solvents: (a) snapshots of final state of three binary mixtures showing only *n*-C_28_ and (b) weight fraction of the waxy alkane found in the solvent phase as a function of simulation time. The vertical line indicates the point at which steady state is assumed (2.5 × 10^5^ DPD time units) except for *n*-C_15_ + *n*-C_20_, which is taken to be 4.5 × 10^5^ DPD time units.

Figure 6 shows the weight fraction of heavy alkane (wf_2_) in the solvent phase as a function of time. In the initial stages of the simulation some of the heavy alkane dissolves into the solvent, leading to a rapid increase of wf_2_ from a starting point of zero, while the remainder solidifies and becomes ordered and static. We found that in most cases, it took approximately 2.5 × 10^5^ DPD time units before the solute concentration reached equilibrium (i.e., a constant concentration of heavy alkane in solvent) and around 4.5 × 10^5^ DPD time units for the *n*-C_15_ + *n*-C_20_ system because the relatively high solubility of the wax (*n*-C_20_) in the solvent (*n*-C_15_) means that this system takes considerably longer to equilibrate. We sampled the solute volume fraction, ϕ_2_, over the last 1.5 × 10^4^ DPD time units to obtain final estimates of the equilibrium solubility, and these results are compared to the experiment in Table 5. The solubilities of the sampled heavy alkanes in most of the solvents is small (< 0.1), and as such (given the simulated volume), only a small number of molecules may be present in the solvent phase at any one time. Thus, it may be difficult to match accurately experiments from the simulation. Nevertheless, the value for *n*-C_7_ + *n*-C_28_ is consistent with that found in our earlier study^22^ and corresponds to a mole fraction of 0.027, where experimentally it is around 0.025. We find qualitatively these models rank the solubilities in the correct order, with the longer alkanes being more insoluble than the shorter ones, and all are within a factor of 2 of the experimental value. Some of the results give very close matches, such as M + *n*-C_32_. The worst match C_2_B + *n*-C_32_ is 6 times too small. Overall, this method shows promise for directly predicting solubilities from the simulation.

**Table 5:** Predicted and Experimental Solubilities for Waxy Alkanes in Aromatic Solvents, by Volume Fraction^a^

solvent + solute	ϕ_expt_^32^	ϕ_model_	rel. error
*n*-C_15_ + *n*-C_20_	0.405	0.2994 (0.0055)	0.26
*n*-C_7_ + *n*-C_28_	0.065	0.0853 (0.0059)	0.31
*n*-C_7_ + *n*-C_32_	0.020	0.0372 (0.0066)	0.86
B + *n-C*_28_	0.0585	0.0127 (0.0012)	–0.14
T + *n-C*_28_	0.0738	0.1569 (0.0022)	–0.78
M + *n-C*_28_	0.0669	0.0349 (0.0013)	–0.48
C_2_B + *n-C*_28_	0.0547	0.0285 (0.0413)	–0.48
B + *n-C*_32_	0.0126	0.0065 (0.0020)	–0.48
T + *n-C*_32_	0.0177	0.0308 (0.0045)	0.74
M + *n-C*_32_	0.0184	0.0170 (0.0042)	0.78
C_2_B + *n-C*_32_	0.0130	0.0021 (0.0005)	–1.01

a Standard deviation given in parentheses.

### 3.10 Behavior of Linear Diphenylalkanes

Having successfully modeled the behavior of alkylbenzenes, we finally studied diphenylalkanes of the form BC_*n*_B as a kind of stress-test of the model. We examined diphenylmethane (BCB) and dibenzyl (BC_2_B) to 1,18-diphenyloctadecane (BC_18_B). No adjustment was made to the DPD interaction parameters when running these simulations. All of these materials are experimentally observed to be solid at room temperatures due to their high melting points. However, none of our models solidified (SI), suggesting a limitation of the current DPD methodology, specifically, that the solidification seen so far is waxy in nature and driven by the long linear alkyl chain length.

In the SI, we considered three areas of improvement for the model: additional restriction of molecular conformations by introduction of dihedral constraints, extending the model to include electrostatic contributions (in this case introducing a dipole on the aromatic ring), and introducing chemical specificity into the pairwise thermostat by making γ_*ij*_ dependent on chemical identity (this last one should leave the equilibrium state untouched but may facilitate its appearance by promoting certain nucleation and growth pathways).

We find that each is able to influence the MSD measured for the molecule, but spatial ordering is largely unaffected until a small MSD was observed, a feat only observed when using electrostatics. Hence, we conclude that the crystallization of the shorter-length diphenylalkane molecules may require the inclusion of electrostatic forces on the benzene ring that encourage regular alignments between molecules (such as introducing a dipole moment to the aromatic ring), but we do not extensively test this in the article. Alternatively, it may be possible to represent the directionality of the short-ranged electrostatic interactions by short-range orientational interactions or similar attractive forces.

As fine tuning such a model would require development of improved electrostatic models for DPD (incorporating partial charges and dipoles, as discussed in our other work on static charges in surfactants^23^), the development of this concept is considered beyond the scope of the current article.

## 4 Discussion and Conclusions

Studying the alkylbenzene family has enabled us to explore two key observations that can be made from observing the DPD literature, namely that authors appear to prefer to adopt models with a limited number of bead types and that partial charges are not included in DPD models. This may lie in the origins of DPD where beads represented large fragments of molecules (e.g., blocks in a block co-polymer), but as the DPD literature has developed, the resolution has increased significantly (where a bead will often represent a chemical group). We have shown that the DPD model is able to achieve a high level of accuracy but only when a sufficient amount of detailed chemistry is captured such as realistic bond length and bond angle constants. Furthermore, we have shown that at this relatively fine level of coarse-graining, we cannot adopt a simple molecular fragmentation approach (i.e., universal bead types), but instead, we require an approach similar to the more sophisticated atomistic models wherein the bead interactions are dependent on both the specific molecular fragments and the neighboring chemistry. For example, we have demonstrated that a benzene ring cannot be modeled using only a single bead type if substitutions are to be made onto the ring. That is to say, the bead types used for benzene (e.g., *a*CHCH bead, in this study) must be different to those used for the phenyl/benzyl group (*a*CHC) to reflect the local chemical environment. By extension, several variants of the *a*CHC bead type exist depending on the length of the alkyl side chain (or other groups) such that the use of a single bead type on its own cannot correctly match all the data.

This begs the question: Why are specialized beads necessary? We believe that the answer lies in a point already touched upon in discussing the liquid densities of longer-chain alkylbenzenes, namely that with the chosen degree of coarse-graining, the typical bond lengths *R*_0_^*ij*^ are ≲ 0.5 *r*_c_ , much smaller than the typical non-bonded interaction ranges *R_ij_* ≈ *r*_c_. Thus, the actual repulsion between beads can be much stronger than that expected from the “bare” pairwise interactions because the bonded neighbors also overlap. By using specialized beads, we are able to compensate for this many-body effect.

As discussed in our work on pure alkanes,^22^ an alternative could be to adopt a multi-component many-body DPD approach and allow the pairwise interactions to become dependent on the local environment.^15,39,40^ However, such an approach introduces a proliferation of parameters, which is not obviously advantageous compared to adding specialized bead types. Of course, with many-body DPD, one also has the potential to reproduce vapor–liquid phase coexistence,^41−45^ which is not possible in the present class of models with purely repulsive non-bonded interactions.^46^

Using appropriate bond and angle constraints, we are able to capture the freezing transition as a function of chain length (i.e., wax formation at room temperature) for the longer alkylbenzenes containing one benzyl group per molecule. However, the freezing of diphenylalkanes containing two benzyl groups per molecule proves difficult to reproduce. This suggests that the model is only able to capture ordering caused by geometrical packing constraints such as that driven by alkyl backbone alignment, and some crucial additional physics is currently missing from the model.

We postulate that this limitation may be overcome by some consideration of the electrostatics (dipole and π–π attractions) of the benzyl ring, which is not captured in the current model (which is also true of other existing coarse-grain models that do not include electrostatics). Incorporating explicit electrostatics into the model would provide its own challenges as decisions would need to be made on the best way to distribute the charge across the aromatic ring (which may prove to be unique to the molecule structure, hence limiting the transferability of bead types between molecules) and the choice of electrostatic representation (i.e., the use of a global relative permittivity, typical in DPD, is problematic as the value depends on the medium such that oil is very different to water and thus limits transferability across formulations). Additionally, the use of electrostatics would significantly increase the computational cost of the simulation. As such, it may be better to instead use a short-range conservative attractive force or orientational interaction to mimic the directionality of the short-range electrostatic interaction.

Nevertheless, the effect is much less prevalent in the linear alkylbenzenes (i.e., with only one aromatic group), which are less dominated by ring interactions, and here, the model does a better job at replicating wax formation as a function of chain length since it deviates from reality by only two carbons. Furthermore, we anticipate similar difficulties in matching melting points for other polar(izable) molecules. Therefore, a parameterization strategy that simultaneously fits the repulsion amplitudes, *A_ij_*, plus any partial charges to, *inter alia*, liquid and solid phase densities, dipole moments, and melting points may be required. Such an approach would obviously benefit from the judicious deployment of machine learning methods and automated fitting algorithms.

## Supporting Information Available

Further details on how the DPD models were fitted and validated; raw statistics produced from simulation and their comparison to the experiment; confirmation of the insensitivity to system size; performance of model containing no specialized bead types; performance of models using a scaled interaction parameter; performance of the model for neat diphenylalkane and possible methods for its improvement (PDF)
