# Supplementary material for: Modeling Alkyl Aromatic Hydrocarbons with Dissipative Particle Dynamics
Source: J Phys Chem B. 2022 Jul 7;126(28):5351–61. doi: 10.1021/acs.jpcb.2c02048 (PMC9310027; doi:10.1021/acs.jpcb.2c02048)
Supplement: Supplementary file 1 — jp2c02048_si_001.pdf [file jp2c02048_si_001.pdf]

Supporting Information: Modeling Alkyl Aromatic Hydrocarbons with Dissipative Particle Dynamics

David J. Bray,*,† Richard L. Anderson,† Patrick B. Warren,† and Kenneth Lewtas‡,¶

† *The Hartree Centre, STFC Daresbury Laboratory, Warrington, WA4 4AD, United Kingdom*

‡*Lewtas Science & Technologies Ltd., 246 Banbury Road, Oxford, OX2 7DY, United Kingdom*

¶*School of Chemistry, The University of Edinburgh, Joseph Black Building, David Brewster Road, Edinburgh, EH9 3FJ, United Kingdom.*

E-mail: david.bray@stfc.ac.uk

A Obtaining Bond and Angle Potential Constants

A.1 Generating Reference Structures

Initial atomistic model structures were created, using the Molefacture Plugin in VMD,[1,2] for benzene (B), propylbenzene (C_3B) and heptylbenzene (C_7B). The experimental bond lengths were taken to be: 1.54Å for C(sp^3)−C(sp^3) (*i.e.* intra-alkyl); 1.46Å for C(sp)−C(sp^3) (*i.e.* benzene-alkyl); 1.40Å for C(sp)−C(sp) (*i.e.* intra-benzene); 1.10Å for C(sp^3)−H (*i.e.* intra-alkyl) and 1.08Å for C(sp)−H (*i.e.* intra-benzene). The experimental bond angle was assigned to be 109° for X−C−X (*i.e.* alkyl), 120° for C=C−X (*i.e.* intra-benzene), where X is either C or H atom. All alkyl groups were assumed to be in *trans* conformation, *i.e.* a dihedral angle of 180° for C−C−C−C. Alkyl side chains projecting away from a benzene ring were initially assumed to have a a dihedral angle of 90° for C=C−C−C (note, this is later refined using simulations).

A.2 Estimating Equilibrium Bond Lengths and Bond Angles

From these atomistic models the centre of mass of each DPD bead was calculated and the distance and angles between specific connected DPD beads calculated. The measured bond length between DPD beads was converted into DPD units and then rationalized into a limited set of values. For example, subtle variations in lengths and angles of benzene ring (depending on combination of *a*CHC, *a*CHCH or *a*CHCH) are rationalised to a single value such that in DPD the geometry of the triangular benzene ring (of three beads) remains the same irrespective of the combination of beads.

A.3 Validating Bond Stiffness and Rigidity Using Atomistic Simulations

Atomistic molecular dynamic (MD) simulations of ethylbenzene (C_2B) and butylbenzene (C_4B), hexylbenzene (C_6B) were run using Gromacs 2018.4[3] whilst adopting the OPLS all-atom force-field.[4] Each simulation contained 1000 molecules and were run under NPT conditions (of 298.15 K and 1 atm) until the simulation reached equilibration (defined as stable density). At each sampled

time frame, the centre of mass of each *virtual* coarse-grained bead is obtained, derived from the atomistic coordinates represented by the bead, and the appropriate *bond* length or *bond* angle measured. These angles and bond lengths are then used to calculate the time-averaged distribution (probability density function).

Next, we modeled the equivalent molecule *via* DPD simulation and calculated the angle and bond length distributions directly from the bead coordinates of each molecule. To allow comparison we convert all DPD distances into real units using the distance conversion of 5.65Å.[5]

B Raw Statistics Produced From Simulation

For densities, where possible we use available literature data on liquid phase densities. Where the alkylbenzene is solid at room temperature we adopt the measured value for the undercooled liquid. For each molecule the experimental melting point is used to determine whether the physical state at 298 K and 1 atm should be (crystalline) solid or liquid.

B.1 Pure Benzene, Small Benzene Derivatives and Alkylbenzene

Tables S1 and S2 give the raw statistics obtained from simulations using the final parameter set outlined in the main text. Measurements reported were taken between 10×10^3 and 20×10^3 DPD time units. All simulations contain 6000 molecules.

Table S1 Comparison Between Experiment and Model Densities and State for Neat Benzene and Small Benzene Derivatives.

T_m is the experimental melting point and MSD the mean square displacement as defined in main text.

Molecule	Formula	Exp. density (g/cm^3)	Sim. density (g/cm^3)	rel. error	Exp. T_m	MSD @500	Sim. state
benzene	C_6H_6	0.873 60[6]	0.872 26	0.15%	6 °C[7]	116.3	liquid
toluene	C_7H_8	0.861 9[8]	0.860 00	0.22%	-95 °C[7]	101.1	liquid
o-xylene	C_8H_{10}	0.875 16[9]	0.873 20	0.22%	-24 °C[7]	84.3	liquid
p-xylene	C_8H_{10}	0.856 54[9]	0.864 94	-0.98%	13 °C[7]	84.9	liquid
m-xylene	C_8H_{10}	0.859 77[6]	0.859 90	-0.01%	-48 °C[7]	92.5	liquid
mesitylene	C_9H_{12}	0.861 06[6]	0.860 94	0.01%	-45 °C[7]	83.8	liquid
ethylbenzene	C_8H_{10}	0.862 0[8]	0.864 50	-0.22%	-95 °C[7]	91.6	liquid
o-diethylbenzene	$C_{10}H_{14}$	0.875 92[8]	0.866 16	1.11%	-31 °C[7]	70.4	liquid
p-diethylbenzene	$C_{10}H_{14}$	0.857 94[8]	0.866 07	-0.95%	-43 °C[7]	65.5	liquid
m-diethylbenzene	$C_{10}H_{14}$	0.859 93[8]	0.863 22	-0.38%	-84 °C[7]	70.5	liquid
1,3,5-triethylbenzene	$C_{12}H_{18}$	0.861 5[8]	0.862 14	-0.07%	-45 °C	52.4	liquid
o-ethyltoluene	C_9H_{12}	0.876 57[7]	0.867 30	1.06%	-81 °C[7]	78.4	liquid
p-ethyltoluene	C_9H_{12}	0.857 02[7]	0.863 70	-0.78%	-62 °C[7]	74.0	liquid
m-ethyltoluene	C_9H_{12}	0.860 4[7]	0.858 50	0.22%	-96 °C[7]	80.1	liquid

Table S2 Comparison Between Experiment and Model Densities and State for Neat Alkylbenzene.

T_m is the experimental melting point, MSD the mean square displacement and S an order parameter as defined in main text.

Molecule	Formula	Exp. density (g/cm^3)	Sim. density (g/cm^3)	rel. error	Exp. T_m	MSD @500	$S \pm 2\sigma$	Sim. state
propylbenzene	C_9H_{12}	0.857 80[7]	0.860 18	-0.28%	-99 °C[7]	76.6	0.05±0.01	liquid
butylbenzene	$C_{10}H_{14}$	0.856 07[7]	0.852 80	0.38%	-88 °C[7]	68.5	0.06±0.01	liquid
pentylbenzene	$C_{11}H_{16}$	0.854 6[7]	0.854 51	0.01%	-75 °C[7]	59.8	0.07±0.01	liquid
hexylbenzene	$C_{12}H_{18}$	0.853 7[7]	0.852 36	0.16%	-61 °C[7]	54.4	0.08±0.01	liquid
heptylbenzene	$C_{13}H_{20}$	0.853 0[7]	0.854 04	-0.12%	-48 °C[7]	48.8	0.09±0.01	liquid
octylbenzene	$C_{14}H_{22}$	0.852 50[7]	0.851 50	0.12%	-36 °C[7]	41.4	0.10±0.01	liquid
nonylbenzene	$C_{15}H_{24}$	0.852 2[7]	0.853 34	-0.13%	-24 °C[7]	38.3	0.12±0.01	liquid
decylbenzene	$C_{16}H_{26}$	0.851 89[7]	0.850 95	0.11%	-14 °C[7]	31.4	0.14±0.01	liquid
undecylbenzene	$C_{17}H_{28}$	0.851 7[7]	0.852 87	-0.14%	-5 °C[7]	28.7	0.15±0.02	liquid
dodecylbenzene	$C_{18}H_{30}$	0.851 6[7]	0.850 49	0.13%	3 °C[7]	23.9	0.19±0.02	liquid
tridecylbenzene	$C_{19}H_{32}$	0.851 5[7]	0.852 25	-0.09%	10 °C[7]	23.0	0.20±0.02	liquid
tetradecylbenzene	$C_{20}H_{34}$	0.851 4[7]	0.850 02	0.16%	16 °C[7]	18.6	0.26±0.02	liquid
pentadecylbenzene	$C_{21}H_{36}$	0.851 3[7]	0.851 74	-0.05%	22 °C[7]	18.3	0.27±0.02	liquid
hexadecylbenzene	$C_{22}H_{38}$	0.851 2[7]†	0.849 53	0.20%	27 °C[7]	14.6	0.42±0.03	(liquid)
heptadecylbenzene	$C_{23}H_{40}$	0.851 2[7]†	0.851 18	0.01%	32 °C[7]	14.7	0.44±0.03	(liquid)
octadecylbenzene	$C_{24}H_{42}$	0.851 1[7]†	0.847 51	0.42%	36 °C[7]	2.2	0.93±0.02	solid
nonadecylbenzene	$C_{25}H_{44}$	0.851 1[7]†	0.848 64	0.31%	40 °C[7]	2.3	0.91±0.04	solid
eicosylbenzene	$C_{26}H_{46}$	0.851 4[7]†	0.847 33	0.48%	44 °C[7]	1.6	0.93±0.01	solid
docosylbenzene	$C_{28}H_{50}$	0.851 0[7]†	0.847 23	0.44%	51 °C[7]	1.3	0.95±0.01	solid
pentacosylbenzene	$C_{31}H_{56}$	0.851 0[7]†	0.848 11	0.34%	59 °C[7]	1.2	0.94±0.01	solid
octacosylbenzene	$C_{34}H_{62}$	0.851 0[7]†	0.846 49	0.53%	66 °C[7]	0.90	0.95±0.01	solid
triacontylbenzene	$C_{36}H_{66}$	0.850 9[7]†	0.846 32	0.54%	70 °C[7]	0.85	0.94±0.01	solid

† density value is for undercooled liquid.

B.2 Miscible Mixtures of Benzene Derivatives and n-Alkanes

Tables S3, S4, S5 and S6 give the raw statistics obtained from the simulations of binary mixtures of benzene derivatives and n-alkanes using the methods and the final parameter set outlined in the

main text. Measurements reported were taken between 10×10^3 and 20×10^3 DPD time units. All simulations contain 24000 beads.

Table S3 Comparison Between Experimental and Model Densities of Miscible Mixtures Using Data From Teja and Rice [10]

Molecule 1	Molecule 2	x_1	Exp. Density (g/cm^3)	Sim. density (g/cm^3)	rel. error
benzene	hexadecane	1	0.874 07	0.872 32	0.20%
benzene	hexadecane	0.9078	0.843 44	0.839 55	0.46%
benzene	hexadecane	0.8018	0.821 27	0.816 76	0.55%
benzene	hexadecane	0.7149	0.808 97	0.804 05	0.61%
benzene	hexadecane	0.6117	0.798 91	0.793 17	0.72%
benzene	hexadecane	0.4962	0.790 95	0.784 38	0.83%
benzene	hexadecane	0.4004	0.784 97	0.778 91	0.77%
benzene	hexadecane	0.3057	0.780 35	0.774 56	0.74%
benzene	hexadecane	0.2035	0.778 35	0.770 86	0.96%
benzene	hexadecane	0	0.772 16	0.765 89	0.81%
benzene	decane	1	0.874 07	0.872 32	0.20%
benzene	decane	0.9018	0.842 70	0.839 00	0.44%
benzene	decane	0.7944	0.815 45	0.811 73	0.46%
benzene	decane	0.7012	0.798 72	0.793 36	0.67%
benzene	decane	0.6074	0.782 95	0.778 26	0.60%
benzene	decane	0.5022	0.768 41	0.764 30	0.53%
benzene	decane	0.4014	0.758 22	0.753 38	0.64%
benzene	decane	0.2923	0.747 82	0.743 42	0.59%
benzene	decane	0.1959	0.740 52	0.736 00	0.61%
benzene	decane	0.1001	0.733 77	0.729 60	0.57%
benzene	decane	0	0.727 34	0.723 83	0.48%
benzene	heptane	1	0.874 07	0.872 32	0.20%
benzene	heptane	0.9026	0.842 20	0.839 99	0.26%
benzene	heptane	0.7974	0.812 91	0.810 73	0.27%
benzene	heptane	0.6973	0.788 80	0.787 20	-0.20%
benzene	heptane	0.5968	0.767 07	0.766 66	-0.05%
benzene	heptane	0.4987	0.748 71	0.749 06	-0.11%
benzene	heptane	0.4041	0.733 12	0.733 94	-0.40%
benzene	heptane	0.3019	0.716 56	0.719 43	-0.32%
benzene	heptane	0.2107	0.705 52	0.707 81	-0.52%
benzene	heptane	0.1064	0.692 30	0.695 91	-0.80%
benzene	heptane	0	0.679 67	0.685 10	-0.92%
benzene	hexane	1	0.874 07	0.872 32	0.20%
benzene	hexane	0.8973	0.841 58	0.838 78	0.33%
benzene	hexane	0.8136	0.816 53	0.814 29	0.27%
benzene	hexane	0.6652	0.772 15	0.776 64	-0.58%
benzene	hexane	0.5709	0.755 44	0.755 73	-0.04%
benzene	hexane	0.5284	0.746 65	0.746 84	-0.03%
benzene	hexane	0.3722	0.715 05	0.717 31	-0.32%
benzene	hexane	0.3163	0.705 12	0.707 75	-0.37%
benzene	hexane	0.2158	0.687 89	0.694 79	-1.00%
benzene	hexane	0.1026	0.670 75	0.675 06	-0.64%
benzene	hexane	0	0.655 36	0.661 14	-0.92%

Table S4 Comparison Between Experimental and Model Densities of Miscible Mixtures Using Data From Asfour *et al.* [8]

Molecule 1	Molecule 2	x_1	Exp. Density (g/cm^3)	Sim. density (g/cm^3)	rel. error
toluene	hexadecane	0.0000	0.770 3	0.765 89	0.57%
toluene	hexadecane	0.06697	0.772 4	0.768 03	0.57%
toluene	hexadecane	0.13099	0.774 5	0.770 39	0.53%
toluene	hexadecane	0.1927	0.776 7	0.772 65	0.52%
toluene	hexadecane	0.2708	0.780 1	0.776 25	0.49%
toluene	hexadecane	0.4333	0.788 5	0.785 33	0.40%
toluene	hexadecane	0.5026	0.792 9	0.790 17	0.34%
toluene	hexadecane	0.5973	0.800 1	0.798 04	0.26%
toluene	hexadecane	0.7017	0.809 9	0.808 69	0.15%
toluene	hexadecane	0.7984	0.821 8	0.821 25	0.07%
toluene	hexadecane	0.89799	0.838 3	0.837 80	0.06%
toluene	hexadecane	1.0000	0.861 9	0.859 99	0.22%
toluene	octane	0.0000	0.698 4	0.698 97	0.08%
toluene	octane	0.04995	0.704 1	0.704 08	0.00%
toluene	octane	0.0910	0.708 7	0.708 61	-0.01%
toluene	octane	0.1548	0.715 8	0.715 95	-0.02%
toluene	octane	0.2566	0.728 1	0.728 44	-0.05%
toluene	octane	0.3472	0.739 9	0.740 39	-0.07%
toluene	octane	0.4447	0.753 8	0.754 35	-0.07%
toluene	octane	0.5437	0.768 97	0.769 86	-0.12%
toluene	octane	0.6408	0.785 4	0.786 29	-0.11%
toluene	octane	0.7612	0.808 1	0.808 69	-0.07%
toluene	octane	0.8747	0.832 2	0.831 94	0.03%
toluene	octane	1.0000	0.861 9	0.859 99	0.22%

Table S5 Comparison Between Experimental and Model Densities of Miscible Mixtures Using Data From Asfour *et al.*[8]

Molecule 1	Molecule 2	x_1	Exp. Density (g/cm^3)	Sim. density (g/cm^3)	rel. error
ethylbenzene	hexadecane	0.0000	0.770 3	0.765 89	0.57%
ethylbenzene	hexadecane	0.0692	0.772 5	0.768 62	0.50%
ethylbenzene	hexadecane	0.1142	0.774 2	0.770 63	0.46%
ethylbenzene	hexadecane	0.2058	0.777 9	0.774 81	0.40%
ethylbenzene	hexadecane	0.3084	0.782 9	0.780 55	0.30%
ethylbenzene	hexadecane	0.4053	0.788 3	0.786 92	0.17%
ethylbenzene	hexadecane	0.5027	0.794 9	0.794 39	0.06%
ethylbenzene	hexadecane	0.6099	0.803 7	0.804 22	-0.06%
ethylbenzene	hexadecane	0.69799	0.812 7	0.813 92	-0.15%
ethylbenzene	hexadecane	0.8029	0.825 7	0.827 92	-0.27%
ethylbenzene	hexadecane	0.9008	0.840 8	0.844 16	-0.40%
ethylbenzene	hexadecane	1.0000	0.862 0	0.864 53	-0.29%
ethylbenzene	octane	0.0000	0.698 4	0.698 97	-0.08%
ethylbenzene	octane	0.0562	0.705 4	0.705 76	-0.05%
ethylbenzene	octane	0.1008	0.710 8	0.711 56	-0.11%
ethylbenzene	octane	0.1983	0.723 6	0.724 53	-0.13%
ethylbenzene	octane	0.2917	0.736 3	0.737 84	-0.21%
ethylbenzene	octane	0.3979	0.751 7	0.753 63	-0.26%
ethylbenzene	octane	0.5898	0.782 3	0.785 12	-0.36%
ethylbenzene	octane	0.7007	0.801 7	0.804 79	-0.38%
ethylbenzene	octane	0.7972	0.819 7	0.823 13	-0.42%
ethylbenzene	octane	0.8964	0.839 6	0.842 95	-0.40%
ethylbenzene	octane	1.0000	0.862 0	0.864 53	-0.29%

Table S6 Comparison Between Experimental and Model Densities of Miscible Mixtures Using Data From Chevalier *et al.*[9], Yang *et al.*[11]

Molecule 1	Molecule 2	x_1	Exp. Density (g/cm^3)	Sim. density (g/cm^3)	rel. error
p-xylene	heptane	0.0000	0.679 9	0.685 10	-0.76%
p-xylene	heptane	0.0948	0.694 32	0.700 32	-0.86%
p-xylene	heptane	0.1911	0.709 39	0.716 35	-0.98%
p-xylene	heptane	0.2880	0.725 04	0.733 04	-1.10%
p-xylene	heptane	0.3865	0.741 5	0.750 71	-1.24%
p-xylene	heptane	0.4856	0.758 45	0.768 95	-1.38%
p-xylene	heptane	0.5861	0.776 51	0.787 84	-1.46%
p-xylene	heptane	0.6877	0.795 16	0.807 38	-1.54%
p-xylene	heptane	0.7906	0.814 74	0.827 01	-1.51%
p-xylene	heptane	0.8949	0.835 24	0.846 52	-1.35%
p-xylene	heptane	1.0000	0.856 7	0.865 12	-0.98%
o-xylene	tetradecane	0.1605	0.768 2	0.765 25	0.38%
o-xylene	tetradecane	0.4252	0.787 8	0.787 28	0.07%
o-xylene	tetradecane	0.5041	0.795 2	0.795 49	-0.04%
o-xylene	tetradecane	0.6743	0.814 7	0.816 59	-0.23%
o-xylene	tetradecane	0.8327	0.839 0	0.841 35	-0.28%

C Testing the Insensitivity to System Size

Figure S1 demonstrates the degree of stability of the result with respect to time and system size for the order parameter S and MSD. In all cases we find the final result is insensitive to the box size used with pentadecylbenzene ($C_{15}B$), hexadecylbenzene ($C_{16}B$) and heptadecylbenzene ($C_{17}B$) being found liquid-like and octadecylbenzene ($C_{18}B$) solid-like.

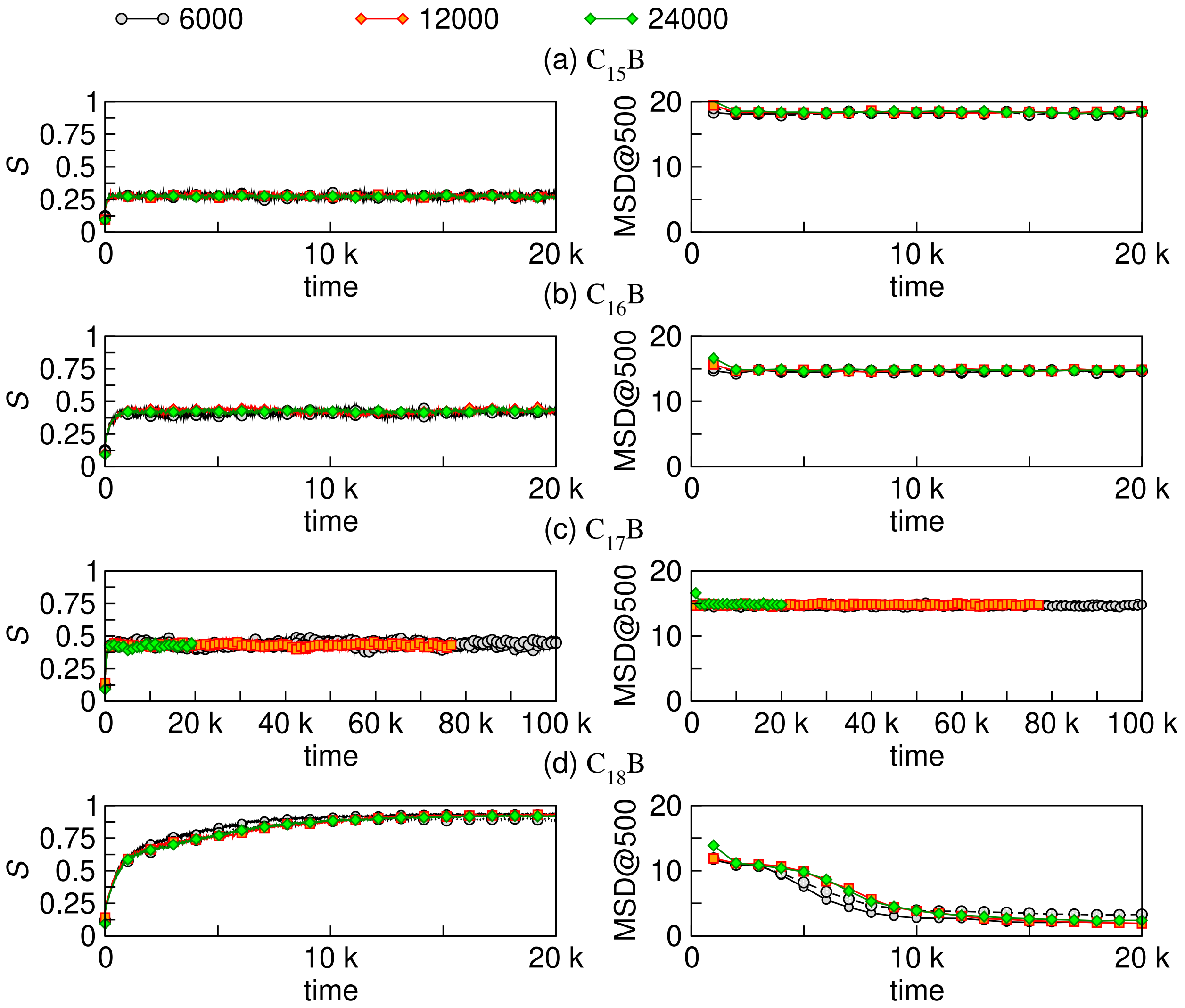

Figure S1 Time dependent behaviour for 4 different neat alkylbenzenes at three system sizes (in number of molecules).

D Performance of Model Containing No Specialised Bead Types

We provide evidence that 'specialised' bead types are needed to account for the chemical behaviour of certain atom group arrangements (such as the side-groups adjacent to the benzene ring). In the following models, all molecules are made from only the alkane (X = CH_3, CH_2CH_2, CH_2) and benzene (*a*CHCH, *a*CHC) bead types using the parameters that have been fitted against benzene and alkanes (*i. e.* A_{ij} of *a*CHC···X are the same as that of *a*CHCH···X and *bn*$CH_m \equiv CH_m$). The bonded structure, bond and angle constraints remain the same as used elsewhere. Table S7 gives the results obtained from these models. As can be seen the accuracy of the density of the small molecules (*e. g.* toluene and mesitylene) are poor with a relative error $> 5\%$ when compared to that achieved for benzene where the relative error was 0.15%. The accuracy improves as the side-chain length increases and the behaviour becomes more dominated by the alkane tail. Nevertheless, even with a C_6 side-chain the accuracy is $\sim 3\%$ and this compares unfavourably to a model that includes specialised beads where the accuracy remains $< 0.5\%$ (See Table S2).

Table S7 Comparison Between Experiment and Model Densities and State for Neat Alkylbenzene When Not Using Specialised Beads (*i. e.* the Simple Model).

MSD is the mean square displacement and *S* an order parameter as defined in main text.

Molecule	Formula	Exp. density (g/cm^3)	Sim. density (g/cm^3)	rel. error	MSD @500	$S \pm 2\sigma$	Sim. State
benzene	C_6H_6	0.873 60[6]	0.872 26	0.15%	116.3	-	liquid
toluene	C_7H_8	0.861 9[8]	0.807 88	6.27%	91.3	-	liquid
mesitylene	C_9H_{12}	0.861 06[6]	0.740 54	14.00%	62.4	-	liquid
ethylbenzene	C_8H_{10}	0.862 0[8]	0.815 68	5.44%	81.5	-	liquid
propylbenzene	C_9H_{12}	0.857 80[7]	0.825 19	3.80%	70.6	0.06±0.01	liquid
butylbenzene	$C_{10}H_{14}$	0.856 07[7]	0.831 53	2.87%	67.2	0.07±0.01	liquid
hexylbenzene	$C_{12}H_{18}$	0.853 7[7]	0.825 51	3.30%	51.5	0.08±0.01	liquid
octylbenzene	$C_{14}H_{22}$	0.852 50[7]	0.828 39	2.83%	38.7	0.11±0.01	liquid
decylbenzene	$C_{16}H_{26}$	0.851 89[7]	0.830 63	2.50%	28.8	0.15±0.01	liquid
dodecylbenzene	$C_{18}H_{30}$	0.851 6[7]	0.832 46	2.25%	22.0	0.20±0.01	liquid
tetradecylbenzene	$C_{20}H_{34}$	0.851 4[7]	0.833 79	2.07%	16.8	0.29±0.01	liquid
hexadecylbenzene	$C_{22}H_{38}$	0.851 2[7]†	0.834 94	1.91%	13.2	0.46±0.05	liquid
octadecylbenzene	$C_{24}H_{42}$	0.851 1[7]†	0.834 99	1.89%	1.7	0.90±0.02	solid
eicosylbenzene	$C_{26}H_{46}$	0.851 4[7]†	0.835 91	1.82%	1.4	0.93±0.01	solid
docosylbenzene	$C_{28}H_{50}$	0.851 0[7]†	0.836 53	1.70%	1.2	0.94±0.01	solid
pentacosylbenzene	$C_{31}H_{56}$	0.851 0[7]†	0.838 41	1.48%	1.1	0.94±0.01	solid
octacosylbenzene	$C_{34}H_{62}$	0.851 0[7]†	0.837 89	1.54%	0.82	0.95±0.01	solid
triacontylbenzene	$C_{36}H_{66}$	0.850 9[7]†	0.838 25	1.49%	0.81	0.06±0.01	solid

† density value is for undercooled liquid.

E Performance of Models Using a Scaled Interaction Parameter

In the main article we describe having to use two sets of A_{ij} interaction parameters for *a*CHC (indicated by *a*CHC^{a} and *a*CHC^{b}) to fit the methyl- / ethylbenzenes (using *a*CHC^{a}) and higher order alkylbenzene C_nB with $n > 2$ (using *a*CHC^{b}). Tables S8 and S9 give additional raw data for models using scale parameter $s = 1.0$ and $\frac{1}{3}$ (equivalent to *a*CHC^{a}) when applied to models of C_nB $n > 2$. When using $s = 1.0$ we obtain an overly dense system consistently $\sim 1\%$ over the experimental value. Using $s = \frac{1}{3}$ produces an under dense system that performs worst at hexylbenzene but improves with longer side-chain lengths and is largely consistent with experiment at lengths $n > 20$ where *a*CHCH···X (X being a non-benzyl alkane bead) interactions become more important.

Table S8 Comparison Between Experiment and Model Densities and State for Neat Alkylbenzene When Using Scaled Ring $s = 1.0$.

MSD is the mean square displacement and *S* an order parameter as defined in main text.

Molecule	Formula	Exp. density (g/cm^3)	Sim. density (g/cm^3)	rel. error	MSD @500	$S \pm 2\sigma$	Sim. State
butylbenzene	$C_{10}H_{14}$	0.856 07[7]	0.839 97	1.88%	64.5	0.06±0.01	liquid
hexylbenzene	$C_{12}H_{18}$	0.853 7[7]	0.839 49	1.66%	50.4	0.08±0.01	liquid
octylbenzene	$C_{14}H_{22}$	0.852 50[7]	0.839 04	1.58%	37.9	0.11±0.01	liquid
decylbenzene	$C_{16}H_{26}$	0.851 89[7]	0.839 23	1.49%	28.3	0.15±0.01	liquid
dodecylbenzene	$C_{18}H_{30}$	0.851 6[7]	0.839 48	1.42%	21.1	0.21±0.01	liquid
tetradecylbenzene	$C_{20}H_{34}$	0.851 4[7]	0.839 74	1.37%	16.2	0.29±0.02	liquid
hexadecylbenzene	$C_{22}H_{38}$	0.851 2[7]†	0.840 37	1.27%	11.9	0.55±0.03	liquid
octadecylbenzene	$C_{24}H_{42}$	0.851 1[7]†	0.841 06	1.18%	2.0	0.91±0.02	solid
eicosylbenzene	$C_{26}H_{46}$	0.851 4[7]†	0.841 21	1.20%	1.2	0.93±0.01	solid
docosylbenzene	$C_{28}H_{50}$	0.851 0[7]†	0.841 38	1.13%	1.1	0.93±0.01	solid
pentacosylbenzene	$C_{31}H_{56}$	0.851 0[7]†	0.843 78	0.97%	0.90	0.94±0.01	solid
octacosylbenzene	$C_{34}H_{62}$	0.851 0[7]†	0.841 58	1.11%	0.79	0.95±0.01	solid
triacontylbenzene	$C_{36}H_{66}$	0.850 9[7]†	0.841 67	1.08%	0.76	0.95±0.01	solid

† density value is for undercooled liquid.

Table S9 Comparison Between Experiment and Model Densities and State for Neat Alkylbenzene When Using Scaled Ring $s = \frac{1}{3}$ (*i.e.* the Parameters of aCHC[a]).

MSD is the mean square displacement and *S* an order parameter as defined in main text.

Molecule	Formula	Exp. density (g/cm^3)	Sim. density (g/cm^3)	rel. error	MSD @500	$S \pm 2\sigma$	Sim. State
butylbenzene	$C_{10}H_{14}$	0.856 07[7]	0.864 79	-1.02%	70.7	0.06±0.01	liquid
hexylbenzene	$C_{12}H_{18}$	0.853 7[7]	0.864 77	-1.30%	56.4	0.08±0.01	liquid
octylbenzene	$C_{14}H_{22}$	0.852 50[7]	0.863 57	-1.30%	43.4	0.10±0.01	liquid
decylbenzene	$C_{16}H_{26}$	0.851 89[7]	0.862 32	-1.22%	33.0	0.13±0.01	liquid
dodecylbenzene	$C_{18}H_{30}$	0.851 6[7]	0.861 11	-1.12%	25.3	0.18±0.02	liquid
tetradecylbenzene	$C_{20}H_{34}$	0.851 4[7]	0.859 96	-1.00%	19.7	0.24±0.02	liquid
hexadecylbenzene	$C_{22}H_{38}$	0.851 2[7]†	0.858 74	-0.89%	15.9	0.36±0.03	liquid
octadecylbenzene	$C_{24}H_{42}$	0.851 1[7]†	0.857 30	-0.73%	16.9	0.83±0.01	liquid
eicosylbenzene	$C_{26}H_{46}$	0.851 4[7]†	0.853 68	-0.27%	2.0	0.91±0.01	solid
docosylbenzene	$C_{28}H_{50}$	0.851 0[7]†	0.853 19	-0.26%	1.7	0.93±0.01	solid
pentacosylbenzene	$C_{31}H_{56}$	0.851 0[7]†	0.853 31	-0.27%	1.5	0.95±0.01	solid
octacosylbenzene	$C_{34}H_{62}$	0.851 0[7]†	0.851 37	-0.04%	1.1	0.95±0.01	solid
triacontylbenzene	$C_{36}H_{66}$	0.850 9[7]†	0.850 97	-0.01%	1.0	0.96±0.01	solid

† density value is for undercooled liquid.

F Performance of the Model for Neat Diphenylalkanes

Table S10 gives the raw statistics obtained from our unmodified model reported in the main article. We note here that it is difficult to obtain experimental data for the diphenylalkane family and are reliant on the limited data given by the manufactures. We find that the MSD reported by most of our models are indicative of liquid behaviour (here, S is less useful as an indicator for small alkyl chain-lengths) and we only find 1,20-diphenyleicosane consistent with being a solid. This transition from liquid to solid is for a similar alkyl chain-length as seen for the alkylbenzenes and implies that solidification is being driven solely by steric constraints of the alkyl chain. In comparison, the available experimental data on the melting point and physical states of diphenylalkanes means that we expect all these models to be solid.

Table S10 Comparison Between Experiment and Model Densities and State for Neat Diphenylalkanes Using Model of Main Text.

T_m is the experimental melting point, MSD the mean square displacement and S an order parameter as defined in main text. All simulations contain 6000 molecules.

Molecule	Formula	Exp. density (g/cm^3)	Sim. density (g/cm^3)	rel. error	Exp. T_m	MSD @500	$S \pm 2\sigma$	Sim. state
diphenylmethane	$C_{13}H_{12}$	1.006‡	0.972 3	3.35%	22-26 °C‡	51.1	-	(liquid)
bibenzyl	$C_{14}H_{14}$	1.014‡	0.999 43	1.44%	52 °C‡	45.5	-	(liquid)
1,3-diphenylpropane	$C_{15}H_{16}$	0.98†	0.992 96	-1.32%		42.9	-	(liquid)
1,4-diphenylbutane	$C_{16}H_{18}$	0.973*	0.973 44	-0.05%	52 °C*	38.2	-	(liquid)
1,6-diphenylhexane	$C_{18}H_{22}$	$\sim$1*	0.956 06	-	78 °C*	31.9	0.10±0.01	(liquid)
1,8-diphenyloctane	$C_{20}H_{26}$		0.942 98	-		26.0	0.12±0.01	(liquid)
1,14-diphenytetradecane	$C_{26}H_{38}$		0.917 48	-		13.2	0.25±0.02	(liquid)
1,16-diphenylhexadecane	$C_{28}H_{42}$		0.911 49	-		10.5	0.34±0.02	(liquid)
1,18-diphenyloctadecane	$C_{30}H_{46}$		0.906 17	-		8.5	0.56±0.03	(liquid)
1,20-diphenyleicosane	$C_{32}H_{50}$		0.900 46	-		3.6	0.86±0.03	solid

† Alfa Aesar manufacture estimate. ‡ Sigma-Aldrich manufacture estimate. * Alfa Chemistry manufacture estimate.
⋆ RSC ChemSpider database.

The above data on the diphenylalkanes suggest that we are unable to correctly capture the solidification of small molecules with the current DPD model. To improve on the model we considered three areas: additional restriction of molecular conformations by introduction of dihedral constraints; introducing chemical specificity into the energy dissipation during collision; extending the model to include electrostatic contributions. In the following sections we explore each in turn to see whether inclusion improves the model. We leave full parameterisation to future work as to increase accuracy further would require reparameterisation of all parameters simultaneously

(including A_{ij}) and can not be done sequentially.

F.1 The Inclusion of Dihedral Constraints Into Model

In the model there are five suitable locations in the molecule structure that are neither co-linear (*i.e.* the alkyl backbone) nor cyclic (*i.e.* within the benzene ring) which might benefit from dihedral constraints, these are the dihedrals made by (i) *a*CHCH$-$*a*CHC$-$*bn*$CH_3$$-$$CH_2CH_2$, (ii) *a*CHCH$-$*a*CHC$-$*bn*$CH_3$$-$$CH_m$, (iii) *a*CHC$-$*bn*$CH_3$$-$$CH_2CH_2$$-$$CH_m$ and (iv) *a*CHC$-$*bn*CH_3 $-$$CH_2CH_2$$-$$CH_2CH_2$ and the improper dihedral *a*CHC($-$*bn*CH_3)($-$*a*CHC(H))$-$*a*CHC(H).

Figure S2 shows the obtain probability distributions for these four dihedrals based on MD simulation (solid line) and the DPD models. When the dihedrals are left to be unconstrained in the DPD model (as in the reported model of the main article) we obtain the dashed line. We can see this is sufficient to broadly capture the *a*CHCH$-$*a*CHC$-$*bn*$CH_3$$-$$CH_m$ and *a*CHC($-$*bn*CH_3) ($-$*a*CHC(H))$-$*a*CHC(H) behaviour as these are captured by the already fairly rigid Benzene ring. However dihderals *a*CHCH$-$*a*CHC$-$*bn*$CH_3$$-$$CH_2CH_2$ and *a*CHC$-$*bn*CH_3 $-$$CH_2CH_2$$-$$CH_2CH_2$ show a much broader range of allowed angles for the DPD model than seen for the MD model and hence are candidates for adding dihedral constraints.

We trialled the harmonic cosine dihedral potential, $\beta U^{D}_{ijkl} = \frac{1}{2} K^{ijkl}_{D} \left(\cos\left(\phi_{ijkl}\right) - \cos\left(\phi^{ijkl}_{0}\right)\right)^{2}$, where K^{ijkl}_{D} is the dihedral constant, ϕ_{ijkl}(in rads) is the dihedral (or improper dihedral) angle resultant from the path based on particles i, j, k, l and ϕ^{ijkl}_{0} is an equilibrium angle based on the chemical identities. We measured the ϕ^{ijkl}_{0} from the all-atom MD simulations and obtained values of 100° for *a*CHCH$-$*a*CHC$-$*bn*CH_3 $-$$CH_2CH_2$, 110° for *a*CHCH$-$*a*CHC$-$*bn*$CH_3$$-$$CH_m$ and 180° for *a*CHC$-$*bn*$CH_3$$-$$CH_2CH_2$ $-$$CH_2CH_2$, respectively. We found that only $K_D \sim 4$ was required for the model to better agree with distributions obtained from atomistic simulation (see dotted line Figure S2). Setting $K_D = 4$ for the dihedrals (i) and (iv) we trialled a DPD model of C_{14}B and obtained a reduced value for MSD@500 (See Table S11) but this was not sufficient to cause the model to solidify.

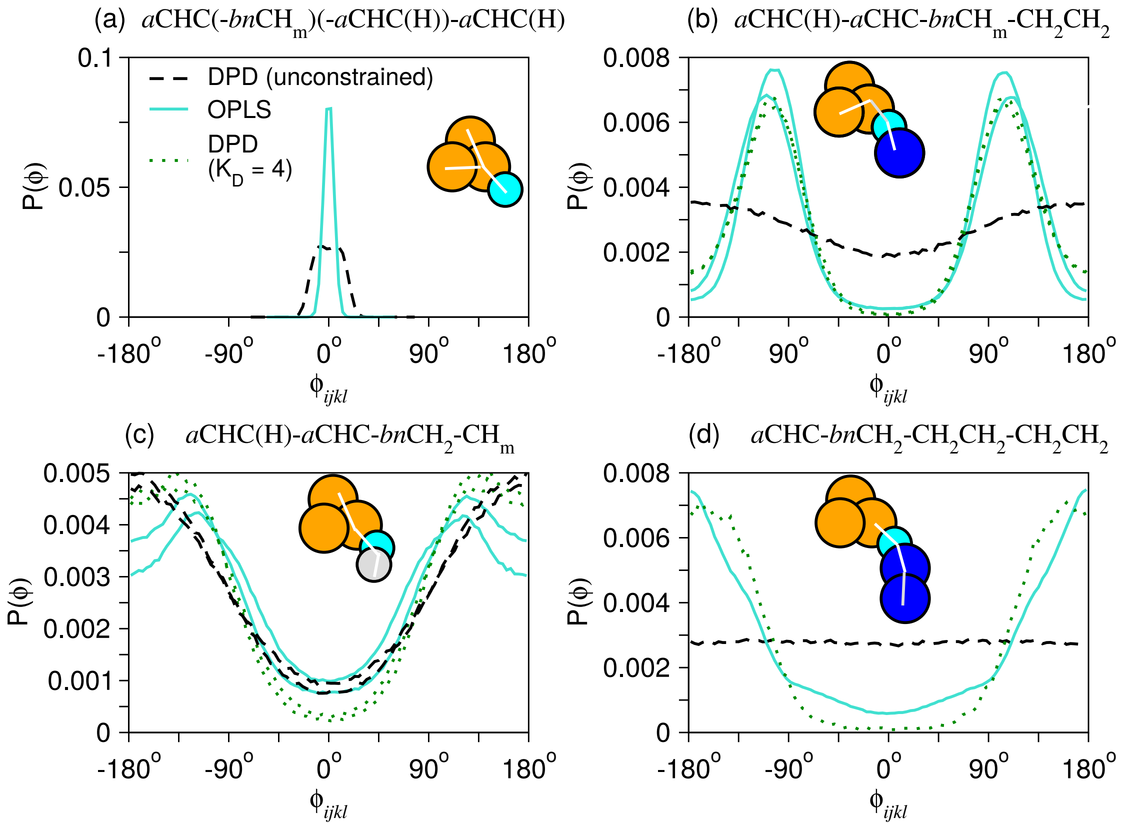

Figure S2 Comparison of the dihedral angle distribution obtained from DPD (dashed/dotted) and MD (solid).

Table S11 Comparison Between Experiment and Model Densities and State for Neat Diphenylalkanes When Including a Dihedral Constraints.

MSD is the mean square displacement and S an order parameter as defined in main text.

K_D	Sim. density (g/cm^3)	rel. error	MSD @500	$S \pm 2\sigma$	Sim. state
		1,14-diphenyltetradecane $C_{26}H_{38}$			
0	0.917 48	-	13.2	0.25±0.02	(liquid)
4*	0.916 72	-	9.9	0.33±0.02	(liquid)

* Requires a smaller time step of $\Delta t = 0.0025$.

F.2 The Inclusion of Chemical Specific γ_{ij} Into Model

We next considered the dissipation term γ_{ij}. In the model of the main article $\gamma_{ij} = 4.5$ for all bead types (as is common throughout the DPD literature). Doing so results in benzene and pen-

tane having comparable MSD@500 valuing 116 and 121, respectively. However, experimentally benzene is three times more viscous than pentane with self-diffusion of 2.27×10^{-5}cm^2/s compared to 5.62×10^{-5}cm^2/s, respectively.[12,13] This motivated us to try using a larger value for $\gamma_{a\text{CHC(H)},a\text{CHC(H)}} \leq 120$ (*i.e.* acting between contacting *a*CHC(H) bead) while keeping $\gamma_{ij} = 4.5$ for the remaining pairs. Table S12 shows the results and we find that we can decrease the diffusion of benzene by a factor of 2.9 (as measured by MSD@500), bibenzyl (BC_1B) by 2.8 and 1,14-diphenyltetradecane ($BC_{14}B$) by 1.6. However, like seen for the dihedral constraints, this is not enough for solidification to occur for $BC_{14}B$ and we do not see any increase in molecule ordering (as seen by S remaining ~ 0.25). We note that by increasing γ_{ij} we reduce the overall density of the system and thus A_{ij} would require adjustment. Never-the-less, correcting for this by reducing A_{ij} for the ring interactions does not significantly change the result.

Table S12 Comparison Between Experiment and Model Densities and State for Neat Diphenylalkanes When Varying $\gamma_{a\text{CHC(H)},a\text{CHC(H)}}$.

MSD is the mean square displacement and S an order parameter as defined in main text.

γ	Sim. density (g/cm^3)	rel. error	MSD @500	$S \pm 2\sigma$	Sim. state
		benzene C_6H_6			
4.5	0.872 26	0.15%	116.3	-	liquid
80	0.839 98	3.85%	61.9	-	liquid
80†	0.870 32	0.37%	65.5	-	liquid
120*	0.860 40	1.51%	40.7	-	liquid
		bibenzyl $C_{14}H_{14}$			
4.5	0.999 43	1.44%	45.5	-	(liquid)
80	0.952 41	6.07%	33.6	-	(liquid)
120*	0.987 65	2.60%	16.4	-	(liquid)
		1,14-diphenyltetradecane $C_{26}H_{38}$			
4.5	0.917 48	-	13.2	0.25±0.02	(liquid)
60	0.912 40	-	10.1	0.24±0.02	(liquid)
80†	0.914 07	-	10.6	0.23±0.01	(liquid)
120*	0.914 02	-	8.2	0.24±0.02	(liquid)

† A_{ij} of self-interactions of ring (aCHC(H)···CHC(H)) modified from 29.5 → 25.5 to better match density. * Requires a smaller time step of $\Delta t = 0.005$.

F.3 The Inclusion of Ring Electrostatics Into Model

To test whether the inclusion of electrostatics (*i. e. via* inclusion of partial charges on the phenyl ring) might drive these systems to solidify we modified the benzene ring by charging the beads *a*CHCH and *bn*$CH_{2/3}$ with a charge $-q$ and $+2q$. The physical motivation of our model is to capture the induced dipole that acts across the benzyl ring for asymmetric molecules (thus a model of benzene, mesitylene would remain uncharged). Note, here our representation is for a single side chain of the benzene ring and a fully generalised model would allow for the ring to vary its partial charge depending on the side groups attached as determined by the experimental dipole moment. Additionally, we have not attempted to fine-tune the charge representation which we leave for future work. Instead this model is used to demonstrate that the inclusion of electrostatics can drive the solidification of these models (in the case of diphenylalkanes) while leaving the state of alkylbenzenes unchanged.

These simulations are performed using the same methodology as for the uncharged. To represent the electrostatic forces we utilised the DPD electrostatic model of González-Melchor *et al.*[14] which assumes a uniform dielectric constant in the simulation box. As these neat systems are not solvated we set the electrostatic coupling parameter to $\Lambda = 1087.408$, *i. e.* equivalent to being in vacuum. Note, this means that Λ is an order of 10 times larger than used in a solvent such as water. To avoid singularities each charge is Slater smeared, a smearing length of 0.929. The electrostatics were solved using a smoothed-particle mesh Ewald (SPME) algorithm,[15] with k-vectors set equal to 30 (*i. e.* greater than the box size) which ensures that the relative error in electrostatic energies was kept below 1%.

We trialled values of q up to 0.5 e for three alkylbenzenes (toluene (T), tetradecylbenzene ($C_{14}B$) and hexadecylbenzene ($C_{16}B$)) and three diphenyl alkanes (bibenzyl (BC_2B), 1,6-diphenylhexane (BC_6B) and 1,14-diphenyltetradecane ($BC_{14}B$)). Table S13 gives the raw statistics for each run.

Figure S3 shows a comparison of the time behaviour for two equivalent alkyl chain-lengths

Table S13 Comparison Between Experiment and Model Densities and State for Models With Electrostatics.

MSD is the mean square displacement and S an order parameter as defined in main text.

charge q (e)	Sim. density (g/cm^3)	rel. error	MSD @500	$S \pm 2\sigma$	Sim. state
**diphenylalkanes**					
		bibenzyl $C_{14}H_{14}$			
0	0.999 43	1.44%	45.5	-	(liquid)
0.1	0.999 85	1.40%	50.3	-	(liquid)
0.2	1.000 33	1.35%	47.2	-	(liquid)
0.3	1.000 77	1.30%	40.1	-	(liquid)
0.4	0.998 16	1.56%	16.3	-	(liquid)
0.5	1.027 99	-1.36%	0.2	-	solid
		1,6-diphenylhexane $C_{18}H_{22}$			
0	0.956 06	-	31.9	0.10±0.01	(liquid)
0.1	0.956 26	-	35.3	0.10±0.01	(liquid)
0.2	0.956 52	-	32.9	0.09±0.01	(liquid)
0.3	0.955 79	-	29.5	0.09±0.01	(liquid)
0.4	0.951 53	-	22.2	0.09±0.01	(liquid)
0.5	0.890 86	-	0.2	0.50±0.02	solid
		1,14-diphenyltetradecane $C_{26}H_{38}$			
0	0.917 48	-	13.2	0.25±0.02	(liquid)
0.1	0.917 25	-	14.4	0.24±0.01	(liquid)
0.2	0.915 60	-	13.2	0.23±0.02	(liquid)
0.3	0.899 48	-	1.2	0.82±0.01	solid
0.4	0.897 81	-	0.3	0.21±0.08	solid
**alkylbenzene**					
		toluene C_7H_8			
0	0.860 00	0.22%	101.1	-	liquid
0.1	0.859 59	0.27%	108.9	-	liquid
0.2	0.859 69	0.26%	107.2	-	liquid
0.3	0.859 32	0.30%	102.0	-	liquid
0.4	0.857 32	0.53%	85.5	-	liquid
0.5	0.831 05	3.56%	36.4	-	liquid
		tetradecylbenzene $C_{20}H_{34}$			
0	0.850 02	0.16%	18.6	0.26±0.02	liquid
0.1	0.850 05	0.16%	20.0	0.25±0.02	liquid
0.2	0.849 79	0.19%	19.1	0.25±0.02	liquid
0.3	0.849 15	0.26%	17.8	0.24±0.02	liquid
0.4	0.847 48	0.46%	15.3	0.24±0.02	liquid
0.5	0.840 07	1.31%	2.9	0.38±0.07	solid
		hexadecylbenzene $C_{22}H_{38}$			
0	0.849 53	0.20%	14.6	0.42±0.03	(liquid)
0.1	0.849 55	0.19%	16.0	0.38±0.02	(liquid)
0.2	0.849 31	0.22%	15.2	0.36±0.02	(liquid)
0.3	0.848 54	0.31%	13.9	0.36±0.02	(liquid)
0.4	0.846 86	0.51%	11.4	0.35±0.02	(liquid)
0.5	0.841 87	1.10%	1.2	0.58±0.06	solid

(C_{14}) for the monophenyl and diphenyl molecular form. At low q the behaviour is very similar to the uncharged model ($q = 0\,e$). However once q is sufficiently large (at $q > 0.4\,e$ (C_{14}B)

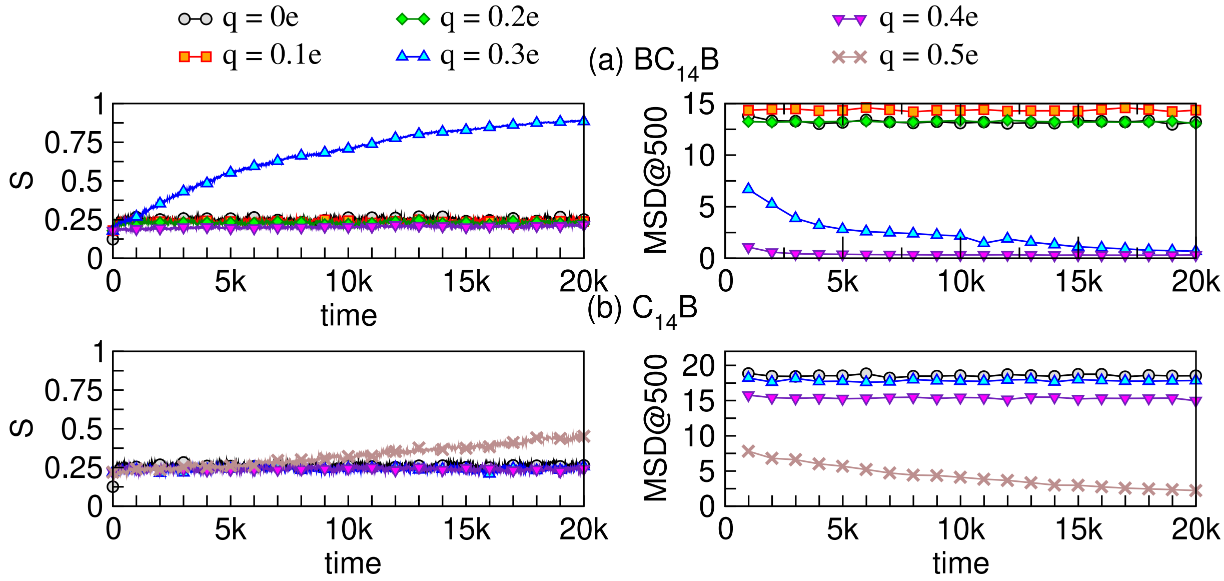

Figure S3 Time dependent behaviour for different charge versions of neat the solution of (a) 1,14-diphenyltetradecane and (b) tetradecylbenzene.

and $q > 0.3$ e ($BC_{14}B$), respectively) the MSD value drops towards zero as time progresses and molecules come out of solution by locking together. This corresponds with an increasing S value with time which doesn't occur for the liquid state (where S remains constant around 0.25). Note, that increasing the charge further makes the molecules lock much more strongly and actually reduces the rate of change of S (*e. g.* for $BC_{14}B$ and $q = 0.4$ e the value of S is actually increasing with time but this is so slow that it barely exceeds 0.25 by the end of the simulation). Figure S4 gives a summary of the final MSD@500 value observed from the run (taken over the final 10k time-units) as a function of q for the six models. We find that the transition from liquid (large MSD) to solid (small MSD) occurs at different values of q for the different models, for example, the transition is q of 0.2-0.3 e for $BC_{14}B$, $q > 0.5$ e for toluene and q of 0.4-0.5 e for the remaining models. Experimentally all apart from $C_{14}B$ and T are solids at room temperature, so fine-tuning a value for q around 0.4-0.5 e may lead to the correct behaviour. We expect the exact value of q at which the transition occurs is dependent on the exact details of the electrostatic model. However it is promising that the values of q are not abnormally large being smaller than 1 e.

In conclusion an improved model that incorporates a combination of chemical specific γ_{ij} (necessary for symmetric rings with no dipole moment, *e. g.* benzene, mesitylene *etc*, to better represent

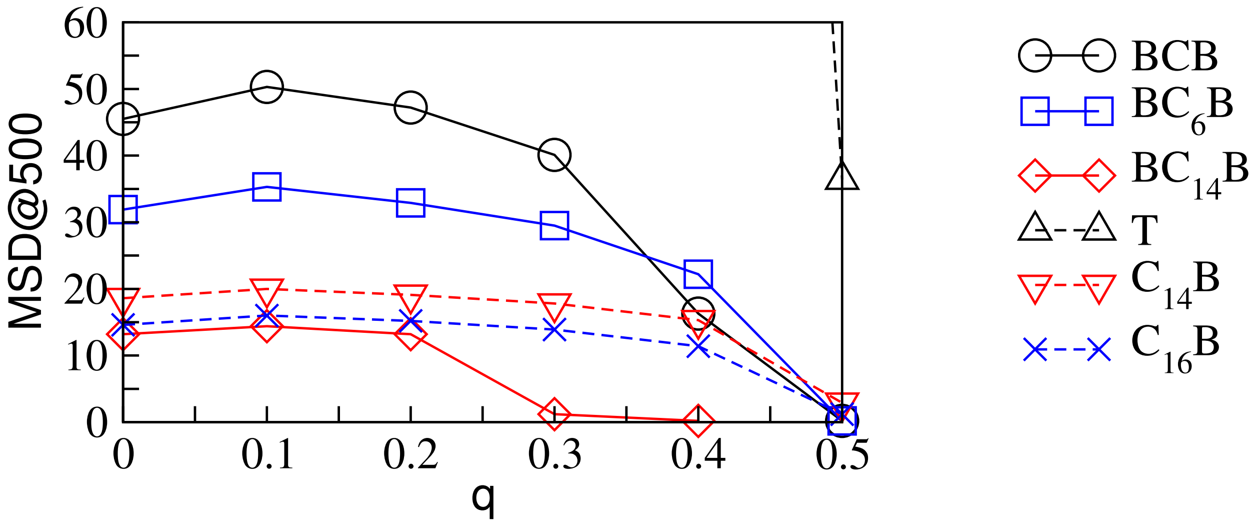

Figure S4 The final time value of MSD@500 for the various models at different values of $q \leq 0.5$. At low q models are in the liquid regime and cross over to solid regime (low MSD) at high q such that we observe a solid state for every model except toluene (T).

self-diffusion rates) and partial charges around the phenyl ring (to capture dipole moments) could offer a way to capture non waxy solidification in DPD. However to do so would necessitate the readjustment of A_{ij} and as such parameterisation of all three (A_{ij}, γ_{ij} and q) would need to be done together.

References

(1) Humphrey, W.; Dalke, A.; Schulten, K. VMD: Visual Molecular Dynamics. *J. Mol. Graphics* **1996**, *14*, 33 – 38.

(2) LLC, M. Molefacture Plugin, Version 1.3. 2012; `www.ks.uiuc.edu/Research/vmd/plugins/molefacture/`.

(3) Spoel, D. V. D.; Lindahl, E.; Hess, B.; Groenhof, G.; Mark, A. E.; Berendsen, H. J. C. GROMACS: Fast, flexible, and free. *J. Comput. Chem.* **2005**, *26*, 1701–1718.

(4) Jorgensen, W. L.; Maxwell, D. S.; Tirado-Rives, J. Development and Testing of the OPLS All-Atom Force Field on Conformational Energetics and Properties of Organic Liquids. *J. Am. Chem. Soc.* **1996**, *118*, 11225–11236.

(5) Anderson, R. L.; Bray, D. J.; Ferrante, A. S.; Noro, M. G.; Stott, I. P.; Warren, P. B. Dissipative Particle Dynamics: Systematic Parametrization Using Water-Octanol Partition Coefficients. *J. Chem. Phys.* **2017**, *147*, 094503.

(6) Al-Kandary, J. A.; Al-Jimaz, A. S.; Abdul-Latif, A.-H. M. Viscosities, Densities, and Speeds of Sound of Binary Mixtures of Benzene, Toluene, o-Xylene, m-Xylene, p-Xylene, and Mesitylene with Anisole at (288.15, 293.15, 298.15, and 303.15) K. *J. Chem. Eng. Data* **2006**, *51*, 2074–2082.

(7) Dreisbach, R. R. *Physical Properties Of Chemical Compounds*; Advances in Chemistry; American Chemical Society, 1961; Vol. 15; Chapter 1, pp 3–523.

(8) Asfour, A. F. A.; Siddique, M. H.; Vavanellos, T. D. Density-Composition Data for Eight Binary Systems Containing Toluene or Ethylbenzene and C8-C16 n-Alkanes at 293.15, 298.15, 308.15, and 313.15 K. *J. Chem. Eng. Data* **1990**, *35*, 192–198.

(9) Chevalier, J. L. E.; Petrino, P. J.; Gaston-Bonhomme, Y. H. Viscosity and Density of Some Aliphatic, Cyclic, and Aromatic Hydrocarbons Binary Liquid Mixtures. *J. Chem. Eng. Data* **1990**, *35*, 206–212.

(10) Teja, A. S.; Rice, P. Densities of Benzene-n-Alkane Mixtures. *J. Chem. Eng. Data* **1976**, *21*, 173–175.

(11) Yang, C.; Xu, W.; Ma, P. Thermodynamic Properties of Binary Mixtures of p-Xylene with Cyclohexane, Heptane, Octane, and N-Methyl-2-pyrrolidone at Several Temperatures. *J. Chem. Eng. Data* **2004**, *49*, 1794–1801.

(12) Falcone, D. R.; Douglass, D. C.; McCall, D. W. Self-Diffusion in Benzene. *J. Phys. Chem.* **1967**, *71*, 2754–2755.

(13) Fishman, E. Self-Diffusion in Liquid Normal Pentane and Normal Heptane. *J. Phys. Chem.* **1955**, *59*, 469–472.

(14) González-Melchor, M.; Mayoral, E.; Velázquez, M. E.; Alejandre, J. Electrostatic Interactions in Dissipative Particle Dynamics Using the Ewald Sums. *J. Chem. Phys.* **2006**, *125*, 224107.

(15) Essmann, U.; Perera, L.; Berkowitz, M. L.; Darden, T.; Lee, H.; Pedersen, L. G. A Smooth Particle Mesh Ewald Method. *J. Chem. Phys.* **1995**, *103*, 8577–8593.
